# Exploring Virome Diversity in Public Data in South America as an Approach for Detecting Viral Sources From Potentially Emerging Viruses

**DOI:** 10.3389/fgene.2021.722857

**Published:** 2022-01-21

**Authors:** Fernando G. Mazur, Leandro M. Morinisi, Junior Olímpio Martins, Pedro Pontes Bueno Guerra, Caio C. M. Freire

**Affiliations:** Department Genetics and Evolution, UFSCar—Federal University of São Carlos, São Carlos, Brazil

**Keywords:** viral diversity, molecular evolution, emerging virus, metagenomic analysis, viral surveillance

## Abstract

The South American continent presents a great diversity of biomes, whose ecosystems are constantly threatened by the expansion of human activity. The emergence and re-emergence of viral populations with impact on the human population and ecosystem have shown increases in the last decades. In deference to the growing accumulation of genomic data, we explore the potential of South American-related public databases to detect signals that contribute to virosphere research. Therefore, our study aims to investigate public databases with emphasis on the surveillance of viruses with medical and ecological relevance. Herein, we profiled 120 “*sequence read archives*” metagenomes from 19 independent projects from the last decade. In a coarse view, our analyses identified only 0.38% of the total number of sequences from viruses, showing a higher proportion of RNA viruses. The metagenomes with the most important viral sequences in the analyzed environmental models were 1) aquatic samples from the Amazon River, 2) sewage from Brasilia, and 3) soil from the state of São Paulo, while the models of animal transmission were detected in mosquitoes from Rio Janeiro and Bats from Amazonia. Also, the classification of viral signals into operational taxonomic units (OTUs) (family) allowed us to infer from metadata a probable host range in the virome detected in each sample analyzed. Further, several motifs and viral sequences are related to specific viruses with emergence potential from *Togaviridae*, *Arenaviridae*, and *Flaviviridae* families. In this context, the exploration of public databases allowed us to evaluate the scope and informative capacity of sequences from third-party public databases and to detect signals related to viruses of clinical or environmental importance, which allowed us to infer traits associated with probable transmission routes or signals of ecological disequilibrium. The evaluation of our results showed that in most cases the size and type of the reference database, the percentage of guanine–cytosine (GC), and the length of the query sequences greatly influence the taxonomic classification of the sequences. In sum, our findings describe how the exploration of public genomic data can be exploited as an approach for epidemiological surveillance and the understanding of the virosphere.

## Introduction

Viral emergencies showed a progressive increase in the last decades (Jones et al., 2008; Gould and Higgs, 2009; Ewald, 2011; Coffey et al., 2014; Nobre et al., 2016). Many factors linked to the expansion of human activity (agriculture, migration, urbanization, etc.) are indicated as agents that threaten the balance of viral communities in nature (Gould and Higgs, 2009; Woolhouse et al., 2012; Mahy, 2014). Currently, accumulated evidence reveals an overwhelming number of new viruses and routes of interaction that were not considered a few decades ago (Wong et al., 2007; Sobel Leonard et al., 2017; Nouri et al., 2018; Schoeman and Fielding, 2019). This impressive volume of new viral sequences allowed us to investigate the still hidden viral diversity that has great magnitude and actively participates in ecological processes (Koonin and Dolja, 2013; Posada-Cespedes and Seifert, 2017). Thus, the sequencing technologies permitted the observation with better resolution of the viral world, changing the old paradigm of parasitic entities to a category containing the largest and most variable genetic information, the virosphere (Shi et al., 2018). Nevertheless, the viral proportions observed to date are only a small fraction of the virosphere volume (Zhang et al., 2018).

Otherwise, the description of viral diversity presents great challenges. These entities have attributes that confer them a rapid response to the environment. Characteristics such as high mutational rate, high reproductive number, recombination, and reorganization, even in very divergent families, contribute to the wide diversity of viral groups (Skewes-Cox et al., 2014; Shi et al., 2016a; Gilbert et al., 2016). Thus, in contrast to microorganism classification methods where it is possible to classify operational taxonomic units (OTUs) from a few markers with high homology (e.g., RNA16S) (Hackl et al., 2004), virus detection is limited by the wide genetic mosaicism detected in the virosphere (Dolja and Koonin, 2018). Therefore, virus or viral trace detection considers alternative strategies to infer the presence of viral activity (Herath et al., 2017). Thus, the diversity discovered in the virosphere, largely explained by horizontal gene transfer (Cai et al., 2006; Gilbert et al., 2016; Wolf et al., 2018), reveals intricate genetic relationships that began to be described with greater resolution in the age of genomics (Xu, 2006).

The increasing addition of new viruses identified in the reference databases, advances in taxonomic classification (Krishnamurthy and Wang, 2017; Simmonds et al., 2017), and the implementation of new statistical and computational techniques make it possible to increase the informative capacity of viral sequences. The combined detection of multiple and diverse patterns in viral genes, such as protein domains or gene sequences of conserved genes, helps to detect OTUs of viral origin despite divergence between taxonomic groups. These innovations make it possible to detect molecular clues of viral origin that may indicate ecological processes, as well as biological phenomena in hosts (Hurst, 2011; Fermin, 2018; Sadeghi et al., 2021). In this sense, the profile of virus species and the abundance of each viral family detected in an organism can reveal key elements in the interaction with its environment. In addition, viral traces can be used as indicators of anomalous bacterial activity, environmental pollutants, or the presence of serious modifications in the ecosystem (Hurst, 2013; Fermin, 2018). This type of evidence allows the detection of large-scale phenomena in the environment and in non-culturable viruses (Roux et al., 2017; Wolf et al., 2020), thus contributing relevant sources of information for ecological monitoring (Fermin, 2018).

The exploration of the world of viral RNA reached a better resolution through the new tools, which permitted the accumulation of enough data to reveal key events in the evolution of this viral group (Duffy et al., 2008). The abundance and characteristics of replication of RNA viruses are favorable in the rapid expansion of its reproductive substrate, which can result in eventual emergencies (Holmes, 2009; Wang and Crameri, 2014). Conjunctions of factors such as diversity disturbances, increased density, and exposure of organisms to sources of infection (Bordería et al., 2011; Geoghegan and Holmes, 2017) are frequently associated with host jumps. However, several viral emergence events resulted from unknown viruses that had reduced sylvatic cycles, due to alterations in the environment, forcing them to occupy new niches and impacting humans (Weaver and Reisen, 2010; Castro et al., 2019). This is the case of several arboviruses and common viruses that infect vertebrates such as *coronaviruses*, *bunyaviruses*, and *retroviruses* (De Wit et al., 2016; Chouin-Carneiro, 2017; Lu et al., 2020). As a surveillance alternative, different research approaches indicate that viral migration pathways can be mapped by observing molecular evidence from viral lineages in a time–space context (Rosario and Breitbart, 2011; Geoghegan and Holmes, 2017).

Moreover, ecological and epidemiological aspects often overlap in the environment. It was indicated that most of the gastrointestinal diseases transmitted by recreational contact on water surfaces have their origin in human waste (Morace et al., 2002; Aw and Gin, 2010; Hjelmsø et al., 2019). Based on this, many countries and research groups have opted for molecular detection methods in aquatic environments as a solution to this problem. It is an important area of surveillance since fecal–oral transmission viruses show a high prevalence in watercourses. As a key feature to consider, fecal–oral transmission viruses show infective capacity at low frequencies (Lin and Ganesh, 2013). Thus, viruses such as *reovirus*, *adenovirus*, *hepatitis*, *rotaviruses* (RV), and human *polyomaviruses* are viral particles of waterborne transmission, which have high stability against environmental perturbations such as high UV degrees and long periods in the environment.

Therefore, the reanalysis of public databases to describe and explore various aspects of the virosphere is an accessible and powerful tool, which is at an early stage. As a first step, this work aims to investigate the large volume of publicly available sequencing data to detect viral sequences. Once viral evidence has been identified, we aim to label, categorize, and contextualize the data, according to the aspect to be analyzed through the collection of suitable metadata. For this purpose, we kept our focus on South America, mainly the Brazilian territory, whose tropical location is associated with the greatest biological diversity. In addition, this area presents a large number of biomes, intense agricultural activity, highly populated cities, and other socioeconomic factors related to the emergence and/or imbalance of viral communities. Depending on the geographical setting and the metagenomes available, our work also evaluates the ability to detect viral signals using the reference viral genomes available to date. In order to do this, we investigated if the knowledge of viral diversity related to a specific context can be used to drive responsible management of human-induced factors with a high impact on the biosphere.

## Materials and Methods

To analyze the presence of viral patterns in public databases, we established a workflow that adapts the heterogeneity of the obtained samples to recover viral sequences using long-established and frequently used tools in sequence similarity (Blast-like) and comparison methods (HMMer-like) with an updated viral database.

### Metagenomic Sequences

We choose metagenomic sequences of samples from Brazil and other countries in South America, using the keywords “Brazil,” “host,” “mosquitoes,” “South America,” and “virus” in the search engine of Sequence Read Archives (“SRA”) database. Also, we filtered the results to obtain sequences from “metagenomic,” “transcriptomic,” “soil,” “Environmental,” “Water,” “Freshwater,” “Host,” and others related to epidemiologically relevant samples.

### Sequence Pre-Processing

The Fastq-dump and Prefetch programs from the SRAtoolkit package v2.3.2 were used to download and manage the files. The integrity of samples was analyzed using FASTQC v0.11.9, and data curation was performed with Fastp (Chen et al., 2018).

### Processing and Analysis of Sequencing Reads

Mapping of viral sequences was performed with Bowtie 2 (Borozan et al., 2013) and the clustered-RVDB-v21 database (Goodacre et al., 2018), which allowed us to index more than 28,000 viruses as the subject for the mapping against the metagenome samples. The results in SAM format were filtered according to PRHED 20 quality, which considers a confidence percentage of 9% chance of error (Suzuki et al., 2017). We also filtered the mapped sequences in which the guanine–cytosine (GC) content ranged from 35% to 75%, using Python and R to perform these analyses. To determine the abundance of viral sequences, we calculated the percentage of reads that mapped with RVDB. Later, we checked the quality of these sequences with Aliview (Larsson, 2014). Once we detected the sequences with the first method (Bowtie 2), the sequences of putative viral origin were translated into the six reading frames (ORF) by EMBOSS (Rice et al., 2000) and subsequently mapped against clustered-RVDB protein (C-RVDB-v21, contains 3,899,699 protein sequences) against Blastp (BLAST+ v2.11.0) using a script based on the MetablastR package.

The results were associated with metadata and taxonomical classification and then filtered, and short reads with low-complexity regions were excluded. Was used clustered-RVDB (C-RVDB-v21) as a database, reporting only viral sequences that aligned with e-value >0.001 and coverage >75% with the level of taxonomic assignment (minimum inclusion criteria = family). Subsequently, we proceeded with a third method, where viral sequences were mapped using hidden Markov model viral profiles with the HMMer tool (Skewes-Cox et al., 2014; Goodacre et al., 2018), which used RVDB-prot (3,899,699) as a reference database to detect viral profiles (vFAMs—13,201 clusters) (Bigot et al., 2019).

### Contig Assembly and Virus Mapping

We performed *de novo* assemblies of the filtered reads with SPADES (Bankevich et al., 2012) and CAP3 (Huang and Madan, 1999). In the same way that we proceeded with the reads, the assembled viral sequences with BLAST+ were inspected, using C-RVDB-v21 database as the first method. Then, the assemblies were filtered, using the same criteria of the reads that were based on GC content, e-value, and coverage. As a second method, each assembled sequence was also translated into the six ORFs by EMBOSS (Rice et al., 2000), analyzed with BLASTP against clustered-RVDB (C-RVDB-v21), and filtered by e-value, coverage, and low level of taxonomic assignment. Finally, contigs were mapped with HMMer using RVDB vFAMs, excluding e-value >0.001.

### Analyses of Results

The results were analyzed using the R language where scripts were created using the packages Gtsummary, phyloR, Gmaps, Strings, SequinR, Biostrings, Rentrez, MetablastR, Pheatmap, and the n Python 3.8.0 language with the Biopython (https://biopython.org/) and pandas (https://pandas.pydata.org/) modules. Correlation analyses of diversity distribution detected by sample were performed according to the type of mapping method. The R packages used in correlation analysis were R, Hmisc, PerformanceAnalytics, and Corrplot packages.

### Analysis of Observed Diversity According to Environmental, Clinical, or Economic Impact

To analyze the host range of sample indicated by the contigs in each sample, metadata information (Fermin, 2018) that shows a taxonomic assignment of hosts in a comprehensive way was used with the following groups of classification: A (Animalia), Ar (Archaea), B (Bacteria), C (Chromista), F (Fungi), P (Plantae), or Pr (Protozoa). In order to identify groups of sequences according to their environmental, economic, veterinary, or clinical impact, our contig sequences were labeled with information from Viral Zone (Hulo et al., 2011). The labeling, taxonomy, and visual representation analyses were performed with the R language using the Rentrez and Sequinr packages. The workflow is shown graphically in Supplementary Figure S1.

In sum, we were able to analyze 19 bioprojects containing 120 SRA experiments, which represented virological diversity from several environments (Supplementary Table S1). In addition, we took into account evidence for, e.g., the presence of families with bioindicator potential, taxonomic groups with high emergence records, or relevant proteins.

## Results

### Viral Sequences

**FIGURE 1:**
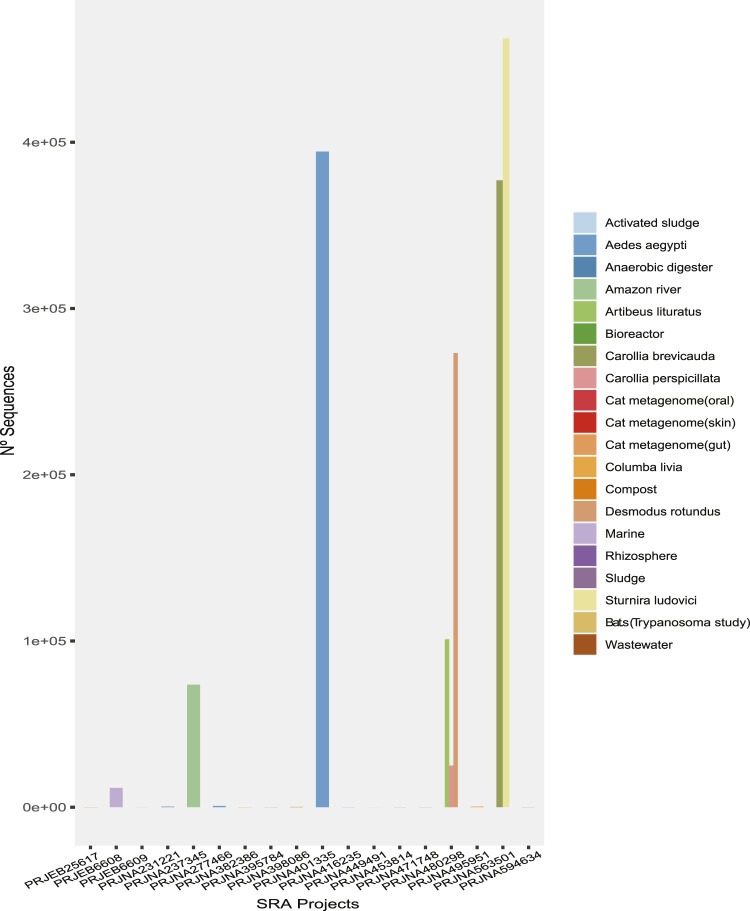
The number of sequences of probable viral origin identified in each metagenome analyzed.

In the first phase of our analysis, 2,750,113 reads from viruses were detected from a total of 7.10E+08 reads in the metagenomes, corresponding to 0.38% of the reads in the 120 metagenomic samples from 19 projects, which represented the heterogeneous sampling origins (Figure 1). Moreover, clustering of the viral sequences by genome type (Table 1) revealed that the highest percentages of virus (Classified) recovered were dsRNA, ssRNA (+), dsDNA, ssRNA (−), and ssRNA-RT (Table 1), in this order.

**TABLE 1:** Summary of rate of viral sequences by genome type.

Genome	Amount (%)
dsDNA	6.8947
dsDNA-RT	0.0001
dsRNA	43.4795
ssDNA (+/−)	0.0104
ssDNA (−)	0.0005
ssRNA-RT	12.1674
ssRNA(+)	17.4409
ssRNA(+/−)	18.6835
ssRNA(−)	1.3227

**FIGURE 2:**
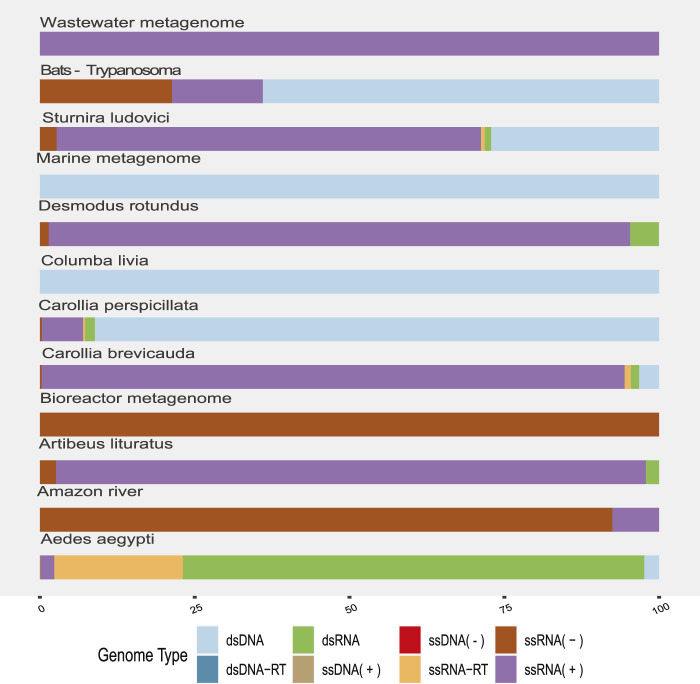
Proportion of viral sequences according to the related genome type by sample origin. The colors allow to identify in the horizontal bars the proportion of sequences clustered according to their genome type (*y*-axis) by sample origin (*x*-axis).

The highest proportion was observed in the dsRNA category from the *Aedes aegypti* samples (Figure 2) and corresponded to sequences identified as *Reoviridae*, *Picobirnaviridae*, *Partitiviridae*, *Hypovirus*, and *Sedoreovirinae*. This uneven distribution of sequences according to the genome in *Aedes* sp. samples could be biased by the methods to obtain and concentrate samples.

RNA (+) viruses were found in more than 21% of the total samples, when they were added to the percentage of other RNA (+) viruses but classified within taxa of variable composition (RNA +/−), totaling more than 40% (Figure 2). In the remaining categories, viruses “ssRNA-RT,” which grouped the *Orthoretrovirinae* and *Retroviridae* families, stood out with 11% and a more homogeneous distribution in the samples. The viral sequences corresponding to ssDNA, dsRNA, and dsDNA-RT classes represented the lowest frequencies that we found. In agreement, we found the highest rate of RNA viruses over DNA in the environment (“Amazonia river,” “Marine,” “Activated sludge,” “Wastewater,” and “Bioreactor”) and bat metagenomes (Figure 2).

**FIGURE 3:**
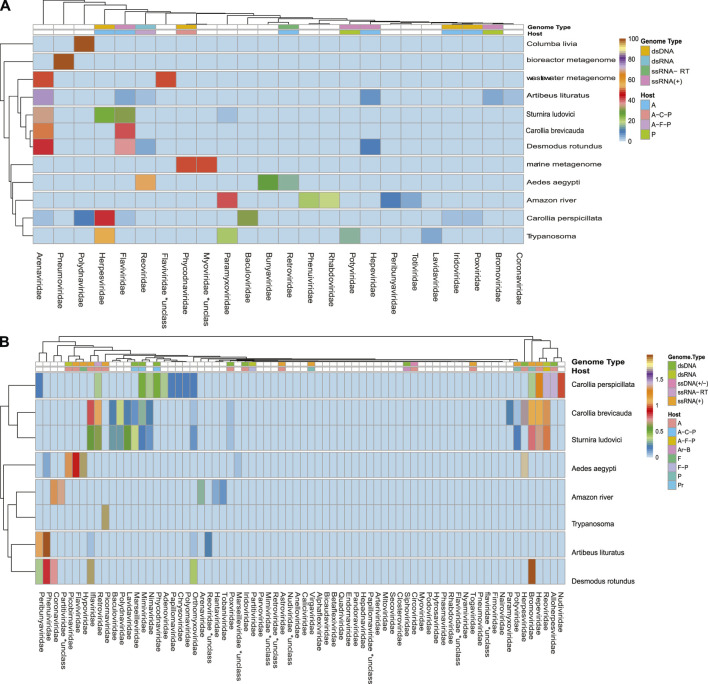
Rate of viral sequences detected in each metagenome. **(A)** The proportions of families recovered with Blastn. **(B)** The proportions of families recovered using contigs translated with Blastp. The RVDB-v21 database reference was used in both methods.

A broad observation of family rate distribution (Figure 3 and Supplementary Figure S4) indicated that most of the viral sequences were grouped in taxonomic categories, which have not yet been clarified. Otherwise, when grouping the sequences considering orders and families with epidemiological relevance (impact on human health or economy), we found a large number of sequences related to RNA (+) families (*Arenaviridae*, *Hepaciviridae*, *Togaviridae*, etc.) and RNA (−) (*Phenuiviridae*, *Peribunyaviridae*, and *Hantaviridae*). The sequences related to relevant dsDNA viruses were mostly classified in the families *Alphaherpesviridae*, *Betaherpesviridae*, *Gamaherpesviridae*, *Picornaviridae*, and *Cytomegalovirus*.

When analyzing the data in a more restrictive way, allowing a minimum level of common taxonomic assignment in the family level, a percentage higher than 50% of all the sequences was indicated to be of viral origin; the methods were described by similarity search (Bowtie 2, and Blastp) and the presence of 78 families in the total of analyzed samples (Figure 3 and Supplementary Figure S4). Likewise, sequences reads and contigs translated into peptides (six ORF) were analyzed with HMMer (Supplementary Figure S9).

An overall evaluation of our results shows that more than 750,000 unique viral sequences were detected in reads (Bowtie 2), 170,000 unique sequences in translated reads (Blastp), 5,800 unique sequences in contigs, and 6,930 unique sequences in translated contigs.

**FIGURE 4:**
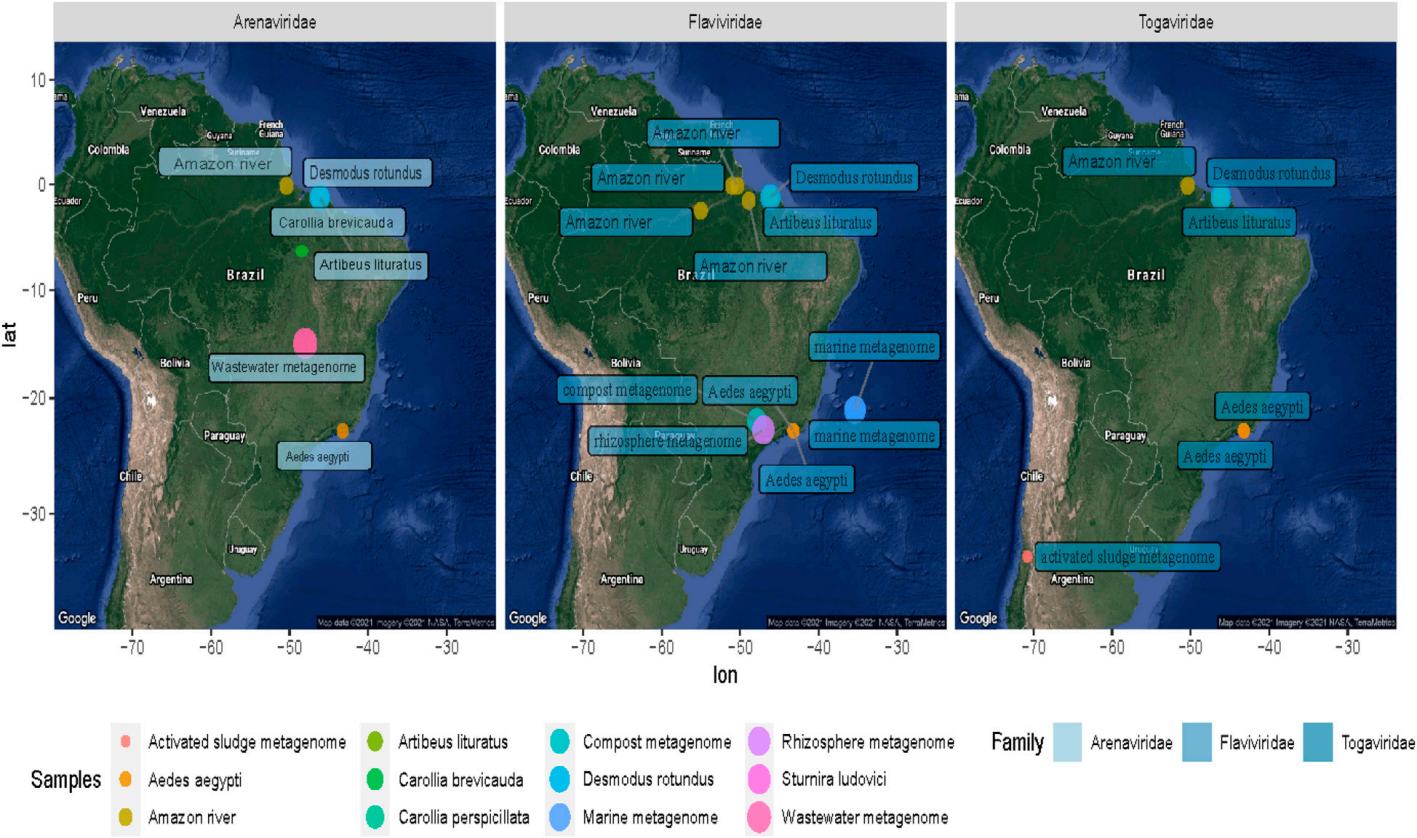
Correlatogram between the assigned family frequency distributions in the metagenomes obtained with all the methods used.

**FIGURE 5:**
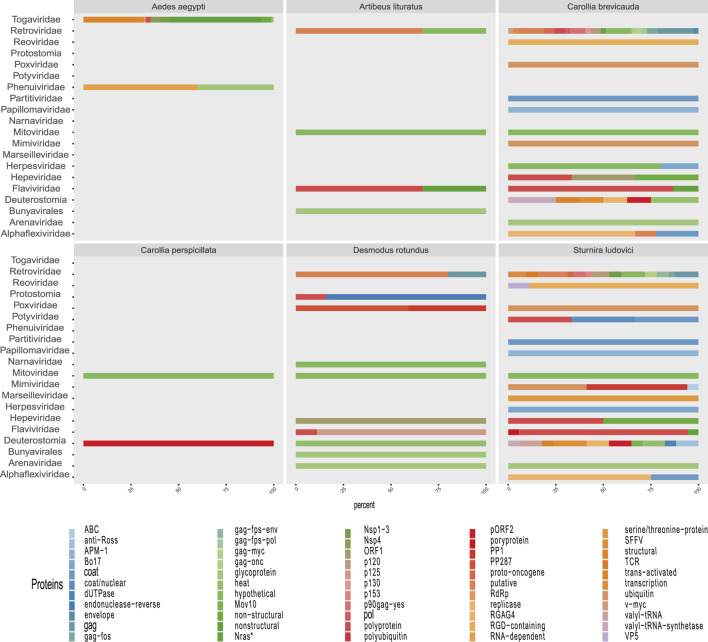
Heatmaps of the proportions of sequencing reads classified into viral families by sample origin. Graphs **(A** and **B)** correspond to the highest proportion (families with >2% of total viral reads by sample) and the lowest proportion of reads (families with <2% of total viral reads by sample). The top columns represent annotations of the genome and host type of each identified family. Uncolored boxes correspond to unclassified families. Genome types are coded as dsDNA, dsRNA, ssDNA(+), ssRNA-RT, and ssRNA(+). Their hosts are coded here as A (Animalia), Ar (Archaea), B (Bacteria), C (Chromista), F (Fungi), and P (Plantae).

**FIGURE 6:**
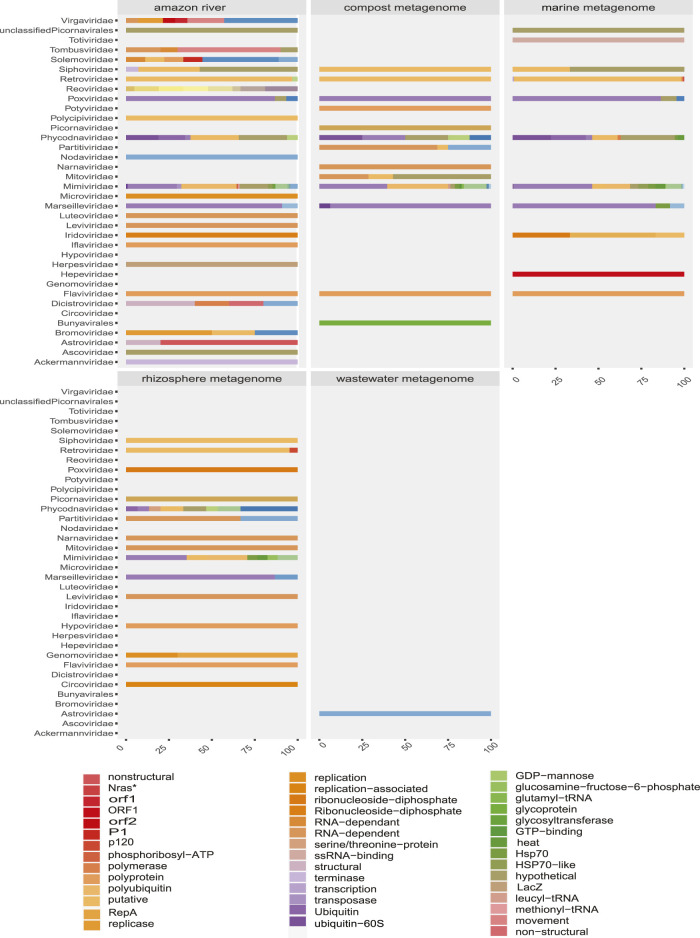
Geographical distribution of the samples containing *Arenavirus*, *Togavirus*, and *Flavivirus*. The colored dots on the map symbolize the coordinates of each sampling group analyzed. The difference in size is proportional to the number of sequences of the family shown.

A more restrictive classification, allowing a minimum assignment criterion to include family, showed that the pairwise alignment and HMM comparison methods indicated similar diversity rates in the samples (Figure 3 and Supplementary Figure S4). Based on this, a correlatogram (Pearson’s correlation) was performed, which included the distribution of viral diversity detected in reads (Supplementary Table S8) and contigs (Supplementary Table S8) with each of the methods (Bowtie, Blastn, Blastp, and HMMer) in each type of sample. The correlogram (Figure 4) revealed a high level of correlation with significance between the distribution of diversity detected among contigs with Blastn, Blastp, and HMMer (pearson cor. >0.98). In the read category, it was possible to detect positive correlation >0.97 between the three methods (Bowtie, Blastp, and HMMer), demonstrating convergence between the diversity detected by pairwise alignment (diversity described by family) and comparison by hidden Markov models (diversity described by vFAMs detected) (Figure 4, Supplementary Figure S9). As expected, the contigs reveal a narrower diversity of families due to the exclusion of sequences in the assembly step, which is reflected in the correlation of their results. However, the lengthening of the sequences by assembly indicated a great diversity of proteins that served to indicate the presence of relevant viral species (Figures 5, 6).

**FIGURE 7:**
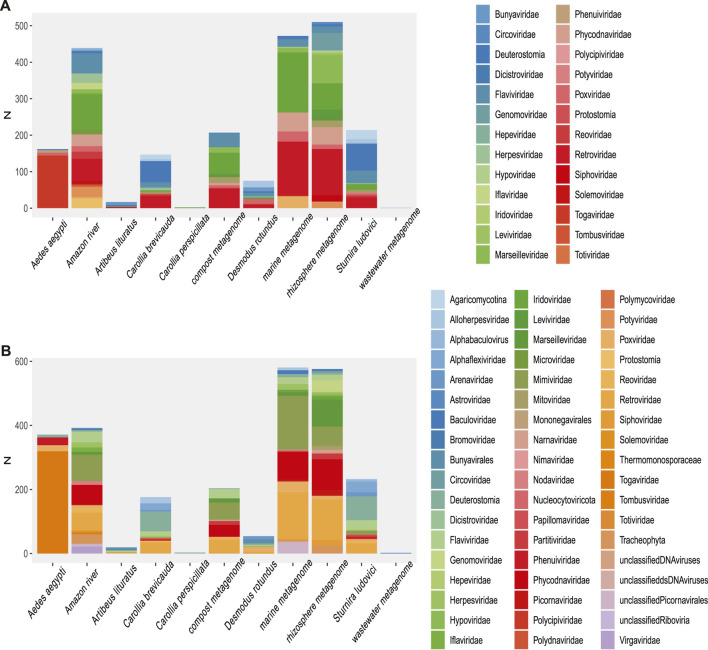
Proportion of contigs obtained related to viral proteins in each metagenome of organisms. The colors allow to identify in the horizontal bars the proportion of sequences related to viral proteins (*x*-axis) according to the viral family (*y*-axis) in each animal model.

**FIGURE 8:**
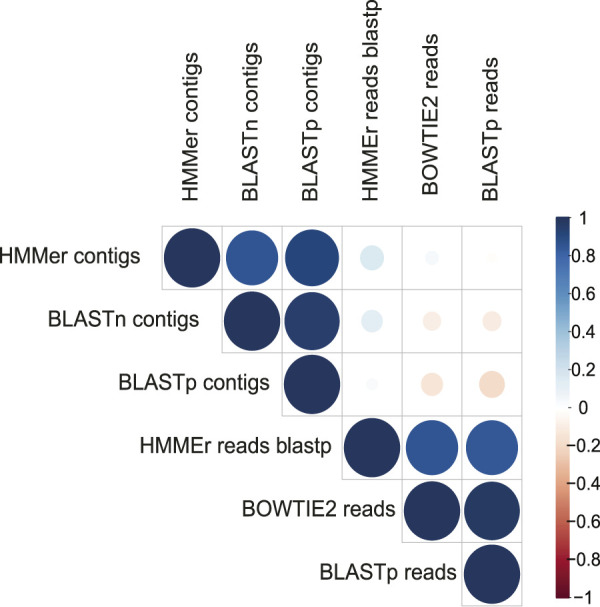
Proportion of contigs obtained related to viral proteins in each environmental metagenome. The colors allow to identify in the horizontal bars the rate of sequences related to viral proteins (*x*-axis) according to the viral family (*y*-axis) in each environmental model.

Some methods detected unique families; likewise, contig assembly in some samples allowed to describe and classify taxa not included by reads. Environmental samples such as rhizosphere, compost, and wastewater metagenome revealed viral sequences only after assembly (Figure 3). A total of 27 common families among reads and translated reads were detected: *Baculoviridae*, *Togaviridae*, *Arenaviridae*, *Phycodnaviridae*, *Reoviridae*, *Mononegavirales*, *Protostomia*, *Retroviridae*, *Herpesviridae*, *Picobirnaviridae*, *Flaviviridae*, *Partitiviridae*, *Mitoviridae*, *Mimiviridae*, *Closteroviridae*, *Picornaviridae*, *Iflaviridae*, *Poxviridae*, *Marseilleviridae*, *Hepeviridae*, *Potyviridae*, *Iridoviridae*, *Astroviridae*, *Caliciviridae*, *Betaflexiviridae*, and *Alphaflexiviridae* (Figure 3 and Supplementary Figure S4). In addition, the mapping of untranslated reads identified 76 families, indicating a significant number of sequences belonging to more than 40 viral families not detected in translated reads (Supplementary Figure S4). The distribution of families detected with contigs by Blastn and Blastp showed no significant differences (Figure 3). Moreover, the assemblers differed in the recovery of some viral species, since the efficiency of CAP3 or SPADEs is probably subject to the sequencing characteristics (transcriptomic, shotgun, pair-end, single-end, etc.) (Rosario and Breitbart, 2011; White et al., 2017). The two assembly approaches recovered in similar proportions of protein classes such as hypothetical (25%), photosystem (18%), nonstructural (8%), and structural (8%). Although no viral sequences larger than 4,000 nt were assembled with the techniques used, it was possible to detect important evidence of viruses such as RDRP, glycoproteins, nucleoproteins, and capsids (Figures 7, 8).

Due to the heterogeneity of the experiments, each sample type detected different levels of viral diversity, which were largely OTUs consistent with the expected viruses according to the type of sample (Figures 3, 5 and Supplementary Figures S4, S5). In addition, many samples analyzed showed a diversity of sequences that allowed classification at the species level.

### Amazon River

In the metagenomes from the Amazon River, a higher amount of sequences from viruses related to the families *Flaviviridae*, *Togaviridae*, and *Paramyxoviridae* were detected. *Flaviviridae* sequences were represented by sequences related to the hepatitis C virus (RNA+). In the *Alphaviridae* group, we detected sequences related to *Semliki Forest virus* (length, ∼200 nt; e-value, 1.10e−66; identity, ∼91%).

In the low proportion (<1% total viral sequences), we identified sequences from *Guanarito mammarenavirus* (length, 300 nt; e-value, 8e−116; identity, ∼96%) and *Phenuiviridae* (*Joao virus*) families (length, ∼300 nt; e-value, 8e−116; identity, ∼96%). In addition, in the lower frequency of reads (<0.1), we were able to observe viral evidence, corresponding to plants (*Betapartitiviurs*, *Bromoviridae*, *Virgaviridae*, *Tobamovirus*, *Tymovirus*, and *Goraviurs*), fungi (*Narnavirus*), and *Enterovirus*, *Paramyxoviridae*, *Bunyaviridae*, *Pestivirus*, *Phenuiviridae*, *Rhaboviridae*, and *Sedoreovirinae*. The assembly also revealed some proteins signals of interesting viral traces, such as movement protein [*Elderberry aureusvirus* 1], VP1 [*Mangshi virus*], movement protein [*Rice virus A*], polymerase [*Reticuloendotheliosis virus*], RNA-dependent RNA polymerase [*Rhizopus microsporus* 23S narnavirus], and nonstructural protein [*Spodoptera exigua* iflavirus 1]. Furthermore, we found sequences indicating a diversity of glycoprotein-specific motifs and structural and nonstructural proteins of *Venezuela equine encephalitis virus* (length, ∼300 nt; e-value, 8e−116; identity, ∼96%) and *Mosso das pedras virus* (length, ∼300 nt; e-value, 8e−116; identity, ∼96%).

### *Aedes aegypti* Samples

The number of viral sequences recovered from *A. aegypti* samples was high as compared with most of the analyzed metagenomes (Supplementary Figure S2). Nonetheless, the highest proportion of viral sequences in *A. aegypti* samples clustered in few viral families. Thus, the largest number of reads was grouped in unclassified families, endogenous elements, and *Retrovirus* sequences. Less frequently, close to 1%, we detected *Flaviviridae*- and *Betaherpesvirinae*-related sequences (Heatmap, Figure 5). The *Flaviviridae* family showed a high occurrence of reads grouped in *Hepacivirus C* or *unclassified Flaviviruses*. Otherwise, the sequences in very low frequency (<0.5%) were grouped in more than 70 groups, which allowed us to point out fragments of epidemiological interest. Also, the highest proportion of RNA (−) viruses were related to viruses like Phasi Charoen virus, a specific insect virus of the *Bunyaviridae* family. In an epidemiological context, our observation points out reads related to *Togaviridae* and fragments that mapped with polyproteins of *Mosso das pedras virus* (length, ∼300; e-value, 6e−73; identity, 100.00%) and *Mucambu virus* (length, 300 nt; e-value, 4e−50; identity, 85%), which are below the *Venezuelan equine Encephalitis virus* complex. In addition, the assembly allowed to detect longer sequences of *Togaviridae* family, corresponding to glycoproteins, SNPs, and structural and nonstructural proteins related to *Venezuelan equine Encephalitis virus*, *Mucambo viru*s, and *Rio Negro virus* (length, 400–1,350; identity, >90%). *Phenuiviridae* was represented by glycoprotein and RDRP of *Phasi Charoen-like phasivirus* (Ferreira et al., 1994; Auguste et al., 2009; Lopes et al., 2014; Espósito and da Fonseca, 2015; Esposito and Fonsecada, 2017). The samples from *Aedes* showed an important rate of RNA viruses; they also indicated a high percentage of DNA sequences, which in most cases correspond to endogenous viruses (Figures 2, 5 and Supplementary Figure S5).

### *Chiroptera* Samples

Our investigations for bat species in public databases revealed six bat metagenomes samples from three projects. The species *Sturnira ludovici* and *Carollia brevicauda*, corresponding to the SRA project PRJNA563501, which focuses on the analysis of vomer-nasal censoring; the project PRJNA480298 that analyzed endogenous viral elements in the species *Desmodus rotundus*, *Carollia perspicillata*, and *Artibeus lituratus* (brain tissue samples); and the project PRJNA382386, which studies the presence of *Trypanosoma* in bat blood samples. First, we observed that the species *S. ludovici* and *C. brevicauda* clustered together, presenting a similar profile in both high and low frequencies. These two species presented a high percentage of *Flavivirus*, *Arenavirus*, *Herpesvirus*, and *Endogenous viral elements*. The remaining species (*D. rotundus*, *C. perspicillata*, and *A. lituratus*), although sampled with the same methodology, showed a different viral profile but overlapping viral families. The diversity profile corresponding to the “Bats (blood)” study was not represented in the heatmap (Figure 5 and Supplementary Figure S5) because the low proportion of reads becomes incomparable when scaling the matrix of viral abundances. In addition, reads grouped in higher proportions showed lower family diversity (N < 4), which contrasted with a higher number of families in lower frequency (N > 8).

The identification of *Arenavirus* in *D. rotundus*, *C. brevicauda*, and *S. ludovici* was also verified by the presence of contigs from these viruses. These sequences corresponded to fragments between 200 and 700 nt of glycoprotein and L-segment regions of *G. mammarenavirus* (e-value, ∼2e−57; identity, 98.46%). These viral motifs were also detected with Blastp in reads and contigs.

### Marine Metagenome

The detected viral sequences were from dsDNA families *Cytomegalovirus*, *Myoviridae*, and *Lymphocryptovirus*. A large number of viral reads from *Prochlorococcus phage P-HM2* were detected (*Myoviridae*) (Supplementary Tables S2–S4).

### Compost and Sugarcane Rhizosphere Metagenomes

Classification of reads pointed to a few viral groups (*Beta Baculoviridae*), *Durnovirales*, and *Herpesvirales*. Nevertheless, the assembly of the sequences revealed the presence of the families *Flaviviridae*, *Mimiviridae*, *Narnaviridae*, *Partitiviridae*, *Poxviridae*, and *Retroviridae* (Supplementary Tables S2, S3). The contigs from *Flaviviridae* (ssRNA+) were related to (length, 300–500 nt; e-value, 7e−39; identity, >90%) polyprotein of *Bovine viral diarrhea virus 2*. *Mimiviridae* (dsDNA) was represented by contigs related to CDS (methionyl-tRNA synthetase, leucyl-tRNA synthetase, glutamyl-tRNA synthetase, cyclophilin type peptidyl-prolyl *cis*-trans, Capsid, Hsp70) of *Edafosvirus*, *Gaeavirus*, *Harvfovirus*, *Terrestrivirus*, and *Hokovirus* HKV1 (length, 150–1,300 nt; e-value, 1e−07; identity, ∼90%). The contigs recovered in *Mitoviridae* corresponded to RDRP proteins, which at the species level were related to *Colletotrichum fructicola* (length, 150–580 nt; e-value, 5e−38; identity, ∼75%), *R. microsporus* (length, 190–450 nt; e-value, 6e−170; identity, ∼95%), and *Pleurotus ostreatus virus* (length, 200–850 nt; e-value, 6e−170; identity, ∼91%), whose hosts are mushrooms. The contigs grouped in *Poxviridae* indicated the presence of polyubiquitins related to *Magpiepox virus*, an avian *Poxivirus* (length, ∼400 nt; e-value, 2e−48; identity, ∼98%). The largest proportion of contigs was grouped in *Retroviridae*, related to the gag protein of *Porcine endogenous retrovirus*.

The second group of samples belongs to the project Metatranscriptome from sugarcane rhizosphere under drought stress, which was collected in February 2015 in São Carlos, Brazil. The analysis in the rhizome samples grouped a low number of sequences, which indicated the presence of *Hypovirus*, *Fabavirus*, and *Retrovirus porcine viruses*.

### Wastewater

Although a low number of sequences could be recovered, we grouped sequences in the medical importance category related to *G. mammarenavirus* (length, 200 nt, 2e−40; identity, 97.06%, *Guanarito Mammarenavirus* isolate CVH-960201 segment L). Also, we detected sequences corresponding to *Astrovirus*, *Norovirus*, and *Oxbow Orthohantavirus* (length, ∼200 nt; identity, ∼86%). The assembly of reads allows recovery sequences of capsids related to human *Astrovirus* (length, ∼190 nt; identity, ∼89.19%).

## Diversity Observed According to Host Range and Environmental and Clinical Impact

**FIGURE 9:**
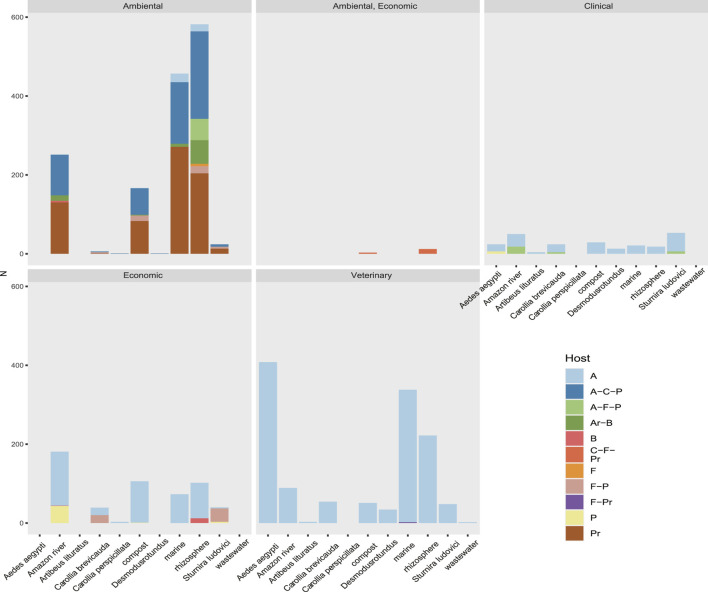
Rate of sequences classified by their relevance clinical, environmental, economic, or veterinary relevance of the taxonomic group to which they belong. Also shown is the host range of the virome of each sample. Their hosts are coded here as A (Animalia), Ar (Archaea), B (Bacteria), C (Chromista), F (Fungi), and P (Plantae).

Using data on host range, ecosystem, and societal impact, we were able to classify the viral sequences and motifs detected in each sample, facilitating the broad observation of groups of interest (Figure 9 and Supplementary Figure S10). The observed clustering was consistent with the expected viral diversity in each sample type, showing in environmental models the highest host potential in “Aquatic metagenome,” “Compost metagenome,” “Marine metagenome,” and “Rizosphere metagenome.” Interestingly, we also detected a great diversity of viruses with environmental impact in samples corresponding to *C. brevicauda* (fruit-eating bat), *S. ludovici* (fruit-eating bat), and *Desmodus rotundus* (Hematophage) (Figure 9). The sequences related to viruses of clinical importance showed a proportional distribution in most of the samples, with a number of viral sequences being identified in “Wastewater metagenomic” and “*C. brevicauda*.” The highest proportion of sequences related to clinical significance was clustered in the Aquatic metagenome and *S. ludovici* samples. The observation of the number of viral sequences according to hosts (Supplementary Figure S10) was consistent with sample type, where the highest number of viral sequences having animals as hosts was detected in the animal model samples. In contrast, viral sequences related to infection in prokaryotes, fungi, bacteria, and plants were mostly detected in Aquatic, Compost, and Rizosphere metagenomes (Figure 9 and Supplementary Figure S10).

## Analysis of Families Detected According to Length of Sequences

Depending on the informative capacity of the sequence, its size can directly influence the classification of viral signals in a metagenome. A histogram based on the length of the sequences identified in reads and contigs a heterogeneous distribution (alternate Supplementary Figures S6, S7) in the frequencies, which in reads is explained by the sequencing methods of the experiment. Reads of Illumina grouped in class intervals between 50 and 200 nt and Ion Torrent greater than 300 nt.

**FIGURE 10:**
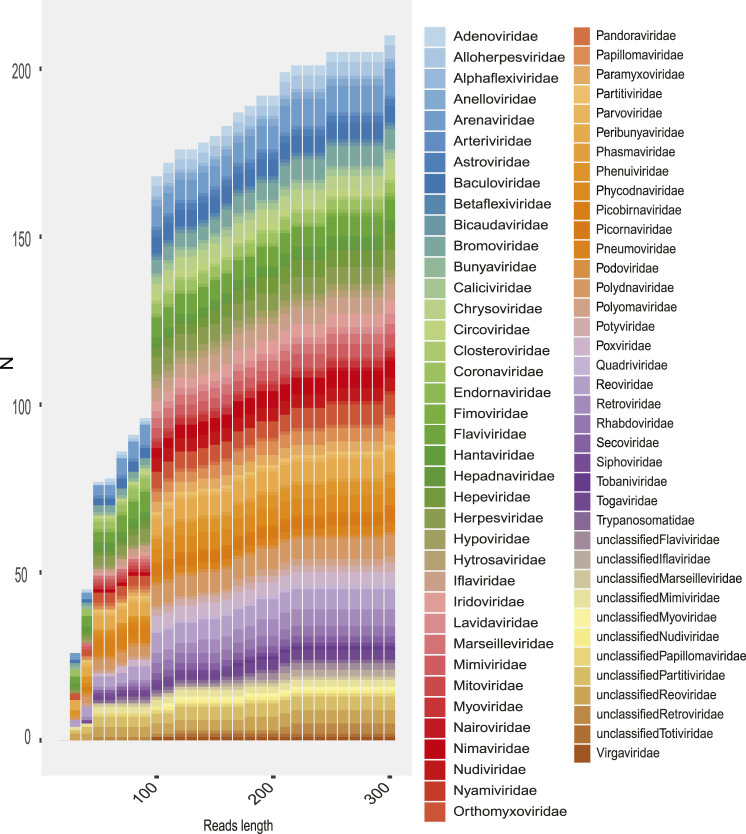
Rate of families recovered in the total metagenomes as the length of reads is increased. The number of families recovered is shown (*y*-axis) according to the length of the sequence (*x*-axis). The colored boxes represent the viral family.

In order to evaluate how the size of the sequences influenced the taxonomic assignment (up to family level), we classified and compared the number of families detected as the length of the reads and contigs increased. We could observe, although to a lesser extent, that short sequences allow the identification of viral families, yet the highest informative capacity to classify is observed from 150 nt (Figure 10). This effect of read size on sequence identification is variable according to the type of experiment (Figure 10), but most indicated greater sensitivity with increasing read length.

## Description of Diversity

The starting point of our analysis focused on the results obtained in reads and contigs processed with Bowtie 2 and Blastn, where we obtained an interesting diversity classification, which was consistent with expected families in the samples. However, because of a large number of ambiguous short sequences that were related with virus, we resorted to analyzing diversity by alternative methods. In addition, a random sampling of sequences was performed to obtain an approximation of probable false positives due to pattern redundancy in nonviral reference proteins. We selected 10,000 random reads and 100 translated contigs (https://github.com/Fernando-Gmzr/Viral-signals-Public-metagenomes) and processed in Blastp to identify sequences related to nonviral origin with a high percentage of identity. The results of the random sampling of reads against reference proteins identified only sequences with a percentage of identity <60%, which belonged to sequences referenced by the RVDB as sequences of *Guanarito* virus (*Arenaviridae*) and *Hepacivirus* (Supplementary Table S6). These sequences were related to mammals and Actinobacteria. Only three contigs identified sequences with a high percentage of identity (proteomic reference of nonviral OTUs), which were related to the mammalian class (Supplementary Table S7).

## Discussion

### Viral Sequences

Virome screening of metagenome samples is limited, mainly by the experimental design of sample collection and sequencing strategies (Breitbart and Rohwer, 2005; Krishnamurthy and Wang, 2017; Obbard, 2018; Metsky et al., 2019). Even studies specifically designed to describe the virome of a sample face challenges that may bias the analyses. Although the viral database is growing, it is still at an early stage and represents only a small portion of the viral dark matter. Thus, sequencing reads are filtered based on homologous sequences, excluding those with the greatest divergence. In this way, the description of the virome in metagenomes is directly related to sequencing advances and the discovery of new viruses (Anthony et al., 2013; Zhang et al., 2019). For example, mimiviruses discovered within the last 2 decades represent a high rate of occurrence in environmental metagenomic samples, which would not be possible without the inclusion of *Mimivirus* genomes in databases.

On the other hand, the experimental design determines to a greater or lesser degree the presence of viral traces, which directly influence the actual composition of the observed virome (Shi et al., 2016b). The experimental design is influenced by important factors such as the exclusion of viral particles by filtering or the inclusion of foreign viruses by contamination (Posada-Cespedes and Seifert, 2017; Fitzpatrick et al., 2021). These limitations are common to all metagenomic analyses but have a greater effect on virome due to viral characteristics. However, metagenomic studies consider the sampling context and the nature of the sample to infer the composition of OTUs. In our study, the metagenomes analyzed whose characteristics were best suited to include sequences of viral origin showed that the most representative viral OTU rates were consistent with the expected composition. Thus, soil and compost samples showed an expected composition, which showed high representativeness of gigantoviruses and viruses related to farm feces. However, some of the metagenomes analyzed in this study, such as marine samples or *Columba livia*, revealed low rates of viral sequences, which are consistent with the type of experiment, sequencing, and nature of the sample. The marine samples revealed a high diversity of proteins, but most were associated with algal and protozoan infecting DNA viruses. In *C. livia*, the only sequences that showed homology with viruses were related to endoviruses, which in many cases are sequences included in the host genome.

A large proportion of metagenomic samples showed the occurrence of viral sequences, with RNA+ viruses as the most representative. Our results showed that of the total mapped reads (0.38%) of 730,253 sequences, 25% of the detected viral sequences corresponded to 64 viruses without full taxonomic classification (Supplementary Table S1). This finding suggested the presence of hidden viral diversity, whose exploration may be restricted by the sampling, methods, and low informative databases. Nevertheless, our results agreed with ecological estimates of viral diversity that reveal that the Baltimore RNA groups dominate the Virosphere eukaryotes, being the RNA+ type the most diverse in this category (Waldron et al., 2018; Wolf et al., 2018).

The results for each sample showed that the diversity detected was limited mainly by the quality and length of the sequences, which are highly dependent on the type of sequencing (Figure 10). In addition, the type of methodology and experimental design are directly related factors, since several samples describing interesting viral diversity were designed to capture transcriptome and virus. The composition of the viral diversity of each sample may show only viral families favored by the sampling methods and in some cases may also include viral OTUs due to contamination. Because of this, the description of viral diversity as an exploratory approach must consider a number of factors such as sample characteristics, metadata associated, families likely to be detected on the sample, method filtering, and the temporal–spatial location of the experiments.

A major limitation in our work was the redundant regions in the sequences, which can contribute to bias in the identification of sequences of viral origin (Marz et al., 2014; Herath et al., 2017; Krishnamurthy and Wang, 2017). Therefore, our approach focused on combining several methods to describe the diversity of each virome. Although each method differentially excluded a considerable proportion of sequences, inspection of reads and contigs with Bowtie 2, Blastn, and Blastp, validated with HMMer by hidden Markov models, confirmed more than 60% of the sequences as of viral origin. Moreover, the correlation between pairwise alignment methods (Bowtie 2 and Blast) and comparison methods (HMMer) revealed interesting results. In addition to the correlation between translated and untranslated reads and contigs, we also observed a correlation with the HMMer results. Unlike similarity methods, HMMer classifies sequences by evaluating their amino acid positional information against viral profiles, which may contain information related to more than one taxonomic family (Skewes-Cox et al., 2014).

The vFAMs are probabilistic models that due to their sensitivity are able to capture remote homologs, extending the informative capacity of the sequences to describe the viral diversity of a sample (Skewes-Cox et al., 2014). This method in combination with pairwise alignment methods proved to be an efficient tool to indicate the viral origin of the sequences. Also, this approach can be used to produce longer sequences or to capture signals from undiscovered viruses, which despite their divergence share patterns in their gene composition (Skewes-Cox et al., 2014; Bzhalava et al., 2018; Bigot et al., 2019).

The convergence in the classification of sequences by both paradigms proved to be efficient in revealing the first layer of viral diversity. The observed signal frequencies make it possible to coarse-view the composition of the virome of each sample (Figures 3, 5, 7, 8; Supplementary Figures S4, S5). In continuation, a more detailed observation shows viral traces of informative relevance in several samples.

### Aedes aegypti

*Aedes* metagenomic samples were obtained from transcriptomics studies, whose focus was on RNAi and dsRNA profile; thus, we were able to detect reads related to dsRNA viruses such as *Reoviridae*, *Picornaviridae*, and *Negeviruses*. Our results corroborated those of other studies, indicating that the virome is constituted in a large proportion by specific insect viruses (Shi et al., 2020) and that the reads recovered in greater proportion corresponded to *Phasi Charoen-like virus* and *Humaita-Tubiacanga virus*, which were described as dominant species (Zakrzewski et al., 2018).

Otherwise, our findings point out the presence of short sequences from genes related to *Venezuelan Equine Encephalitis virus*, *Mayaro virus* complex, and *Venezuelan equine encephalitis virus* complex that belong to *Alphavirus* genus, which comprises viral species that showed emergence events in South America during the last decades (Auguste et al., 2009; Esposito and Fonsecada, 2017). These viruses are single-stranded positive-sense RNA viruses with spherical structures and envelopes that cause febrile diseases with possible encephalitis and arthritis. Although our findings do not represent any news about the circulation of *alphaviruses* in the region, the diversity of proteins found could suggest a probable focus of transmission. Based on this, the recovered sequences reinforce evidence on potentially emerging virus circulation in mosquitoes.

### Amazon River

The detected viral sequences were obtained from quantitative metagenomic and metatranscriptomic analyses from two studies (June 2010 and July 2013) from the Amazon River. In general, the profile of the river samples showed viral evidence from representatives that infect hosts of various domains and kingdoms (Figures 7, 8). Thus, the revealed virome indicated circulation of a variety of infecting viruses of plant-, fungus, and bacteria-, protozoa-, and animal-infecting viruses. The authors have reported that the presence of high amounts of plant viruses in aquatic ecosystems may be associated with fecal contamination or contamination by agricultural activities. Our findings pointed out presented proteins related to species such as *Tropical soda apple mosaic virus*, *Tomato necrotic streak virus*, tomato *Mosaic virus*, *or Rice virus A* (Supplementary Tables S2, S3). Likewise, other sequences corresponding to gag proteins from porcine retrovirus, polyproteins from *Bovine viral diarrhea virus 2*, or proteins from avian *Poxviridae* could suggest transmission routes associated with farms. Although the region of the Amazonian rivers is surrounded by human settlements, our observations are based on low-frequency viral traces, requiring tests for the investigation of pollutants that quantify viral particles with greater precision.

Besides, the virome revealed several viral sequences associated with protozoa and soil bacteria. In this way, giant viruses (*Mimivirus*) were related to *Edafovirus*, *hokovirus*, and *Indivirus* groups. Also, we were able to observe sequences related to insect-specific viruses, such as proteins from *Humaita-Tubiacanga virus* or *Phasi Charoen-like phasivirus*. In the context of aquatic ecosystems, we found evidence of *Phycodnaviridae*, as well as sequences related to photosystem proteins. Infection in algae is a common phenomenon in marine ecosystems where viral activity functions as an important population regulator (Fuhrman, 1999; Fridman et al., 2017). Associated with animal viruses and with emerging potential, we point out with more emphasis evidence of viruses from *Arenavirus* and *Togavirus* families. In this way, these observations correspond to the north of Brazil collected in 2011 and 2013, indicating a broad profile whose traces could suggest probable contamination and a vast spectrum of waterborne viruses. Thus, the “SRA” obtained from these experiments allowed us to describe one of the most interesting results of viral diversity of the present study, which, despite its limitations, managed to reveal viral traces that could facilitate the investigation of ecological processes and transmission networks.

### Marine Metagenome

The observed profiles of marine samples were collected in the Atlantic Ocean, Marine Ecoregion: Trindade and Martin Vaz Islands in 2010-10. The largest number of sequences clustered in photosystem genes (Supplementary Table S3). These genes are responsible for encoding key components of photosynthesis reactions (photosystem II (PSII)). Because of coevolution virus–algae, phage P-HM2 can express photosystems that help to protect its hosts from UV damage, while preventing photoinhibition and upregulating metabolic genes. The viral traces were related to *Prochlorococcus* sp. host, which is a cyanobacteria, considered as a major component of phytoplankton and the main contributor to primary productivity in tropical and subtropical oceans (Fuhrman, 1999; Waldron et al., 2018). The detection of viral evidence related to metabolism is used as an important indicator of viral activity, thereby allowing the detection of abnormal infection rates in important nodes of the ecosystem, such as algae. Due to the methodology used in the collection, the types of sequences expected were related to DNA-type viruses (Aro et al., 1993; Mann et al., 2003). In summary, these results, although they do not reveal the true diversity of viruses present due to the experimental method used, are sufficient evidence to indicate traces of viral activity in algae.

## Soil Samples

This group of samples comes from two metatranscriptomic projects performed in the state of São Paulo, Brazil. The first one corresponds to the compost metatranscriptome, carried out in the city of São Carlos, whose samples were collected at locations 30 cm below the surface. The methodology of this project was designed to evaluate the composting microbial community growing with sugarcane bagasse. The virome profile of each sample was revealed to be consistent with samples associated with soil, plant debris, and fertilizers of animal origin. The largest amount of viral sequences was associated with the genus *Klosneuvirus*. This group, already described as soil giant viruses, possess a genome of ∼1.5 Mb, characterized by hosting aminoacyl transfer RNA synthetases for various amino acids and a great diversity of tRNA-modifying enzymes and translation factors (Schulz et al.). As in the Amazon River samples, the sequences indicated viral traces of *Bovine viral diarrhea virus 2* or proteins associated with porcine *Retroviruses* (Supplementary Tables S2–S4). In this context, these sample results suggest probable exchange pathways that may explain the viral signals.

### Wastewater

Wastewater samples were collected in Brasilia in May 2015 with the objective of monitoring pathogenic microorganisms in the urban environment. Although in a very low quantity, the viral traces identified in wastewater made it possible to reveal the presence of frequently infected *Enteroviruses* in human populations. Crucially, the sequences detected in this metagenome suggest, along with the other *Arenavirus* results, the presence of waterborne transmission.

### Chiroptera

The investigated sequences were obtained from samples from the Brazilian Amazon region in Para state in 2014. The virus profile showed a great diversity of shared families among the analyzed bat species, while some species indicated the presence of unique families. According to a study carried out in 2020, which investigated “The Database of Bat-associated Viruses (DBatVir),” the viral percentages for the most representative families (>1%) were *Coronaviridae* (35%), *Rhabdoviridae* (26%), *Paramyxoviridae* (905%), *Astroviridae* (6.7%), *Adenoviridae* (3.4), *Poluomaviridae* (2.8), *Reoviridae* (2.3%), *Circovirida*e (2.1%), *Herpesviridae* (2.1%), *Flaviviridae* (2.0%), *Picornaviridae* (1.7%), *Parvoviridae* (1.5%), and *Filoviridae* (1.1%) (Letko et al., 2020). Otherwise, our analysis revealed a different viral profile. Although records of coronavirus in bats indicate very high frequency, our study detected very low proportions of coronavirus motifs in bats. Only the species *D. rotundus* and *A. lituratus*, which have different eating habits (hematophagous and fruit-eating), showed the presence of viral patterns related to “Infectious bronchitis virus,” an avian coronavirus. In contrast, families such as *Herpesviridae* and *Flaviviridae* (represented by hepatitis C in our analysis) showed a high frequency of occurrence (Figure 5 and Supplementary Figure S5).

All the ecological characteristics of *Chiroptera* evidence in this group are important vectors for interspecies transmission. Bats comprise a vast group (described as the second largest of mammals), where countless viral exchanges occur, connecting distant taxonomic categories. The wide range of habitats of *Chiroptera* results in the contact and transport of various groups of viruses (Brook and Dobson, 2015; Moratelli and Calisher, 2015). Thus, the coexistence with so many viral families facilitates routes for transmission of plant viruses, mycotic viruses, and vertebrate viruses (Brook and Dobson, 2015; Hayman, 2016). *Arenaviruses*, especially *Mammarenaviruses* in South America, deserve great attention since several countries such as Argentina, Brazil, and Venezuela are endemic regions of the virus that causes hemorrhagic fever (Parodi et al., 1966; Salas et al., 1991; Carroll et al., 2015; Gonzalez et al., 2018). In South America during the last 50 years, outbreaks related to these groups have occurred, resulting in major environmental disturbances (Safronetz et al., 2012; Carroll et al., 2015). Like *Hantavirus*, *Mammarenaviruses* frequently infect rodents and bats (Fernandes et al., 2019). In consequence, viral species that have small and closed cycles in rodents with specific habits, due to environmental alterations, can come into contact with vectors of large distributions, favoring the exposure to humans (Le et al., 2007; Warren and Sawyer, 2019). The occurrence of these factors increases in settlements of low economic stratum, where precarious houses with limited access to electricity and drinking water are favorable for the viral exchange (Jones et al., 2008; Mahy, 2014; Brook and Dobson, 2015; Afelt et al., 2018). This scenario, which frequently occurs as an interface between wild cycles and urban cycles, is a recurring risk factor in South America (Hahn et al., 2014; Nobre et al., 2016; Fernandes et al., 2019; Ellwanger et al., 2020). Due to rapid deforestation and advanced anthropization, the human impact on highly complex ecosystems such as the Amazon rainforest facilitates viral jumps (Afelt et al., 2018; de Mello Malta et al., 2020). In this context, our findings are in agreement with previous records of *Arenavirus* infecting several hosts (Coimbra et al., 1994; de Mello Malta et al., 2020). In sum, the analysis allowed detecting sequences and identifying corresponding viral profiles, where some viral species are common, and others could be specific viral traces of their ecotype, like plant and fungi viruses in frugivorous or *Bovine viral diarrhea virus* in hematophagous (Supplementary Tables S2, S3).

### Biological Correlates of Our Findings

Based on this, the workflow applied in this study allowed us to take advantage of the sequences accumulated in public databases, to reveal in a coarse view the virome of organisms and specific environmental areas. The exploration of metagenomes of mosquitoes from Rio de Janeiro, bats from the Amazon region, or wastewater from Brasilia can serve as data that complement records or guide epidemiological investigations in relevant areas in the future. As a result, the analysis in such heterogeneous samples revealed traces of viral activity in different hosts and environments.

In this scenario, the retrieved tracks point to probable foci of *Alphavirus*, and *Arenavirus* transmission in the Amazon, Brasilia, and Rio de Janeiro regions, which are related to potentially emerging viral groups (Figure 4).

The analysis of third-party public databases as an exploratory approach to virus identification is subject to major challenges and limitations related to the nature of the viruses and to the database that is affected by experimental design and other factors that we cannot control, such as lab contamination. However, the ubiquity and resistance of viruses in the environment, the high reproduction in *foci* of infection, and the strategic choice of transmission sources for monitoring establish viral detection as an important tool. Our findings allowed us to figure out the virus detection and description capability, with reference tools and databases, which are still in their initial stages. Accordingly, it is expected that in the near future, the progressive discovery of new viruses, the optimization of analysis strategies (e.g., hidden Markov models and machine learning), and the exponential accumulation of data will enable new large-scale perspectives to study biodiversity. Consequently, the informative capacity of viral sequences, in addition to being useful epidemiologically, has the potential to contribute to the understanding of the virosphere (Schulz et al., 2018).

## Data Availability

Publicly available datasets were analyzed in this study. These data can be found here: https://github.com/Fernando-GMzr/Viral-signals-Public-metagenomes.

## Supplementary Material

Figure S1 workflow used in the present work to capture viral signals using different methods.

Figure S2 Number spots and viral sequences by sra experiment. On the x-axis are distributed the analyzed experiments where viral sequences were identified. Red bar represents total reads by SRA, Blue bar reads corresponding to viral sequences.

Figure S3 Rate of viral sequences (reads) detected in each metagenome. A) It shows the proportions of families recovered with Bowtie2. B) It shows the proportions of families recovered using reads translated with blastp. In both methods the RVDB-v21 database reference was used.

Figure S4 Geographic distribution of the metagenomes samples. The different colors indicate the type of host or environment from which the sample was extracted.

Figure S5 Frequency histogram of the length of reads retrieved from all metagenomes.

Figure S6 Frequency histogram of the length of translated contigs retrived from all metagenomes.

Figure S7 Heatmaps of the proportions of contigs classified into viral families by sample origin. Graphs A and B correspond to the highest proportion (families with >2% of total viral reads by sample) and lowest proportion of reads (families with <2% of total viral reads by sample). The upper columns represent annotations of the genome and host type of each identified family. Uncolored boxes correspond to unclassified families. Genome types are coded as dsDNA, dsRNA, ssDNA(+), ssRNA-RT, ssRNA(+). Their hosts are coded here as A (Animalia), Ar (Archaea), B (Bacteria), C (Chromista), F (Fungi), P (Plantae).

Figure S8 Range of hosts detected by metagenome type.

Figure S9 Rate of vFAMs detected in reads **(A)** and contigs **(B)** using hiden markov models (HAMMer).

Figure S10 Rate of families recovered per metagenome sample according to the length of reads.

Table S1 Proportion of sequences of viral origin detected in each SRA with their respective associated metadata information.

Table S2 Rate of viral protein signals according to family and type of metagenome analyzed.Also, the table describes functional information of the related proteins.

Table S3 Table of viral signals related to metadata and description of the related protein.

Table S4 Results of contigs processed by blastn. It shows the sequences with their metadata and taxonomical information.

Table S5 Metadata used to classify families by genome type and host.

Table S6 Table of results of random sampling analysis of reads against Blastp using a non-viral database. it shows the sequences with a high percentage of identity (>70%).

Table S7 Table of results of random sampling analysis of contigs against Blastp using a non-viral database. it shows the sequences with a high percentage of identity (>70%).

Table S8 Tables of Distribution of the frequencies obtained by taxonomic assignment (Family) in each sample by pairwise alignment (Bowtie, Blast) and Hidden Markov comparison methods (HMMer).
