# Supplementary figures and images for: Exploring Virome Diversity in Public Data in South America as an Approach for Detecting Viral Sources From Potentially Emerging Viruses
Source: Front Genet. 2022 Jan 21;12:722857. doi: 10.3389/fgene.2021.722857 (PMC8814814; doi:10.3389/fgene.2021.722857)

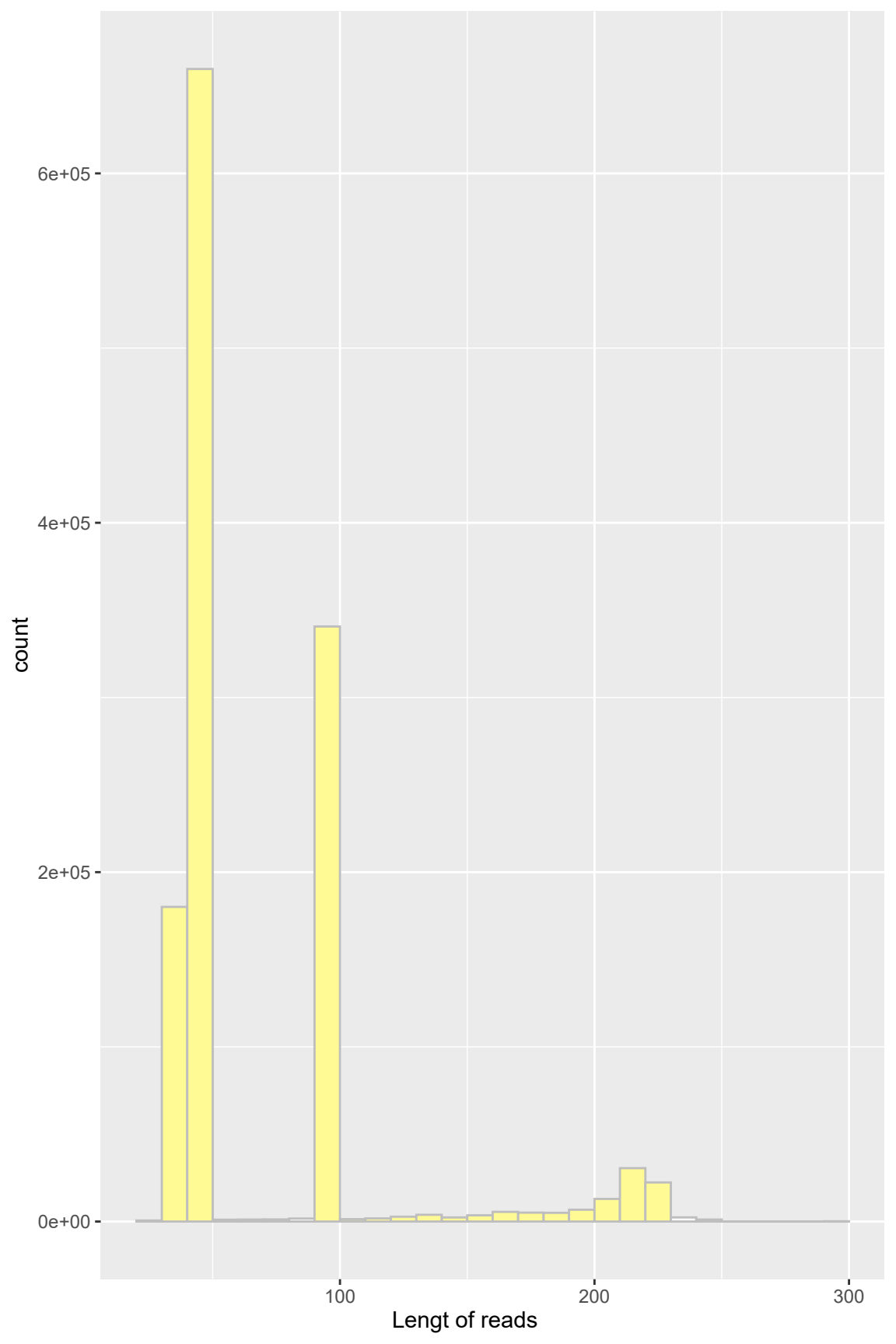

Supplement: Supplementary file 1 [file Image5.pdf]

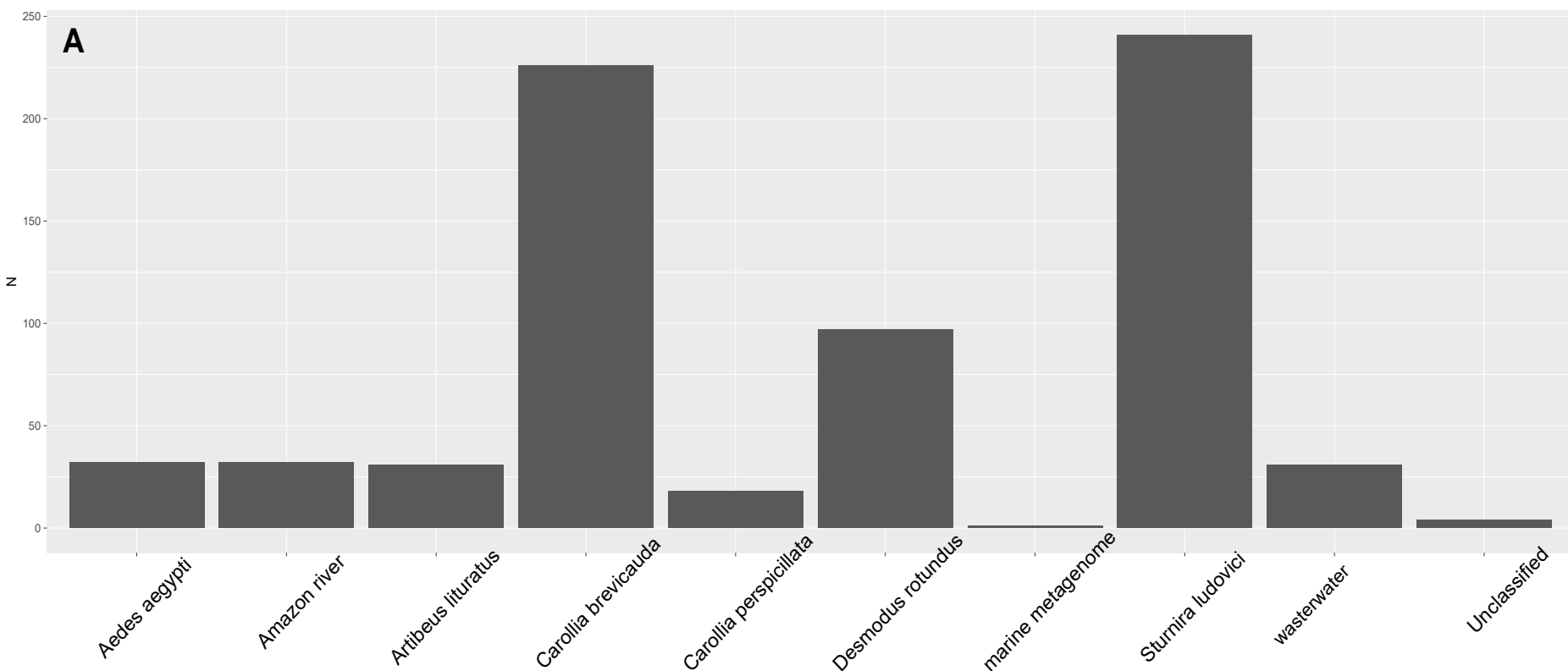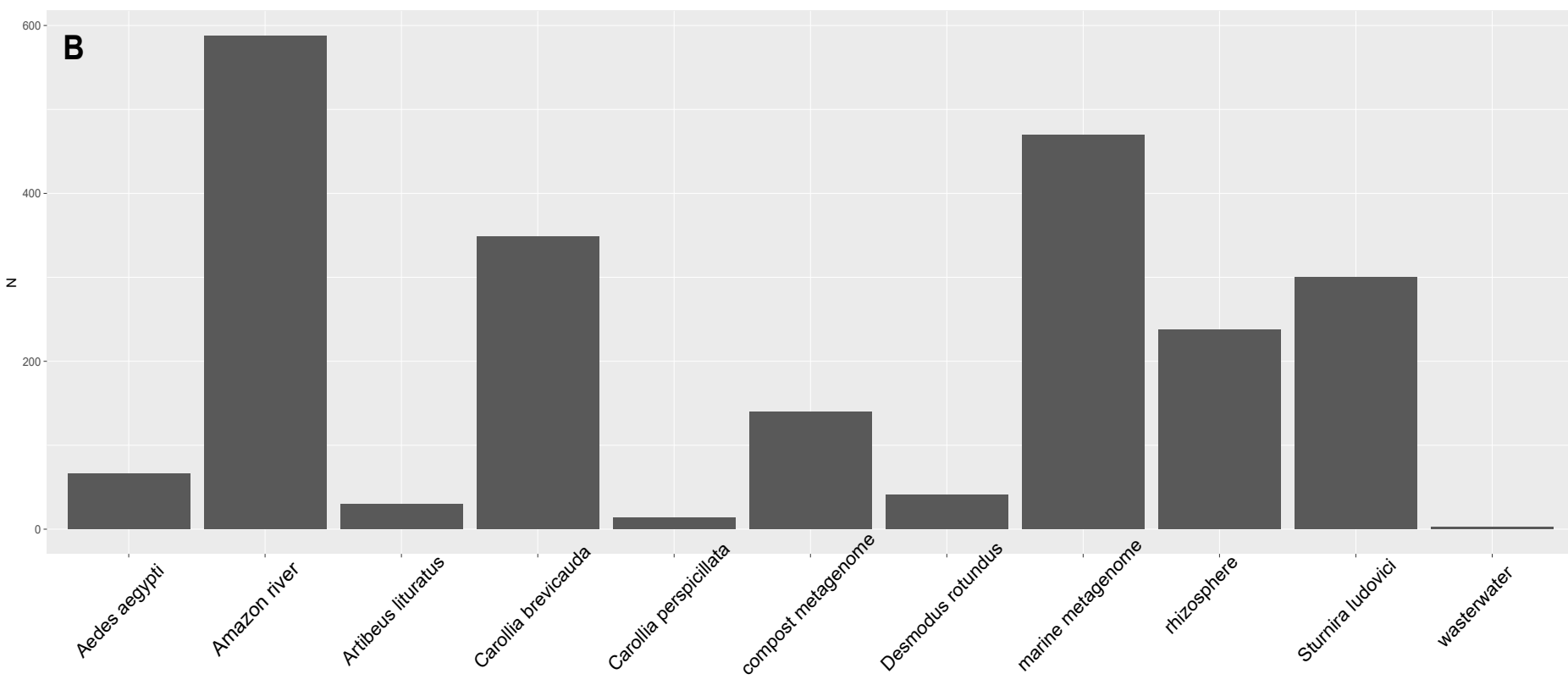

Supplement: Supplementary file 2 [file Image9.pdf]

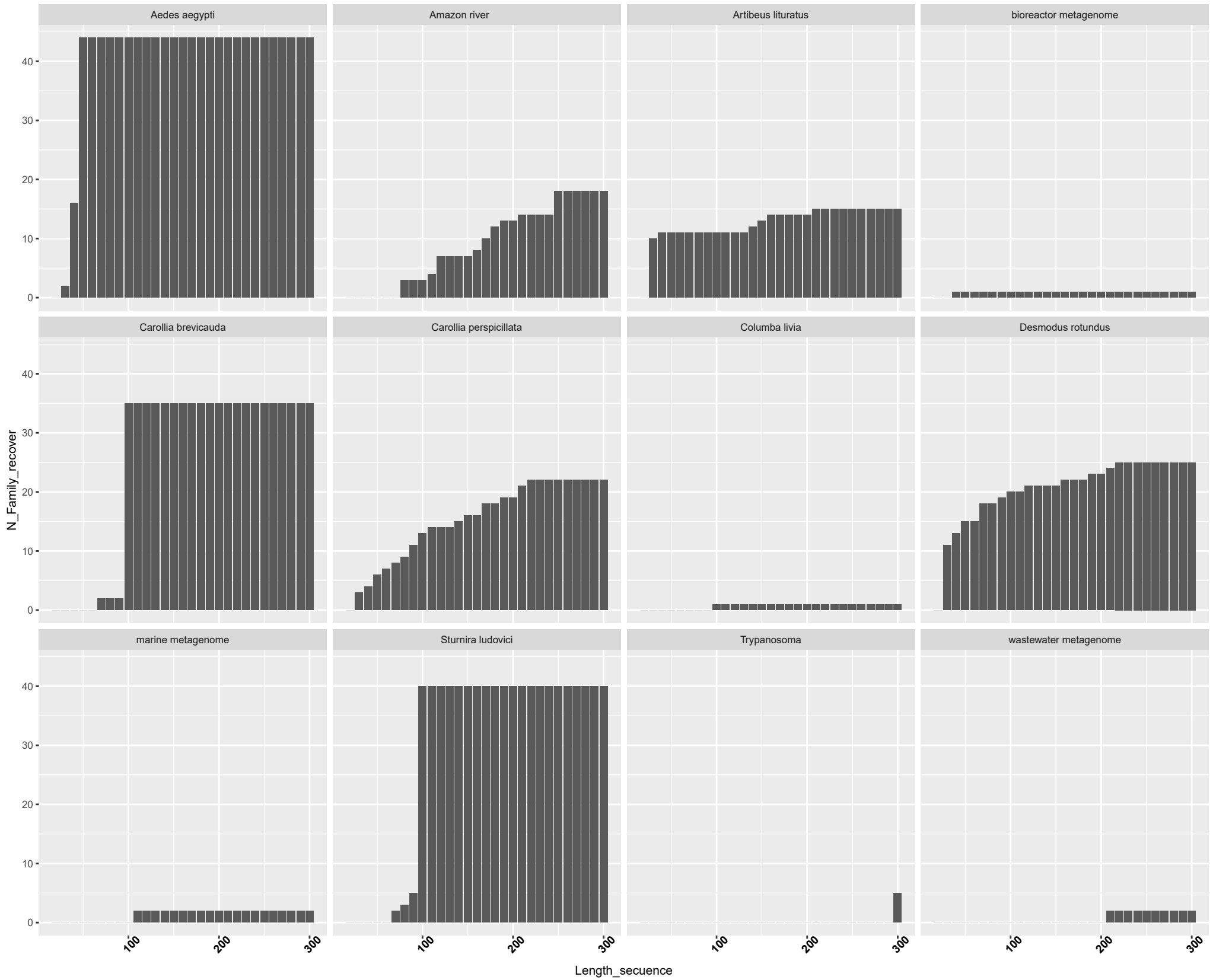

Supplement: Supplementary file 4 [file Image10.pdf]

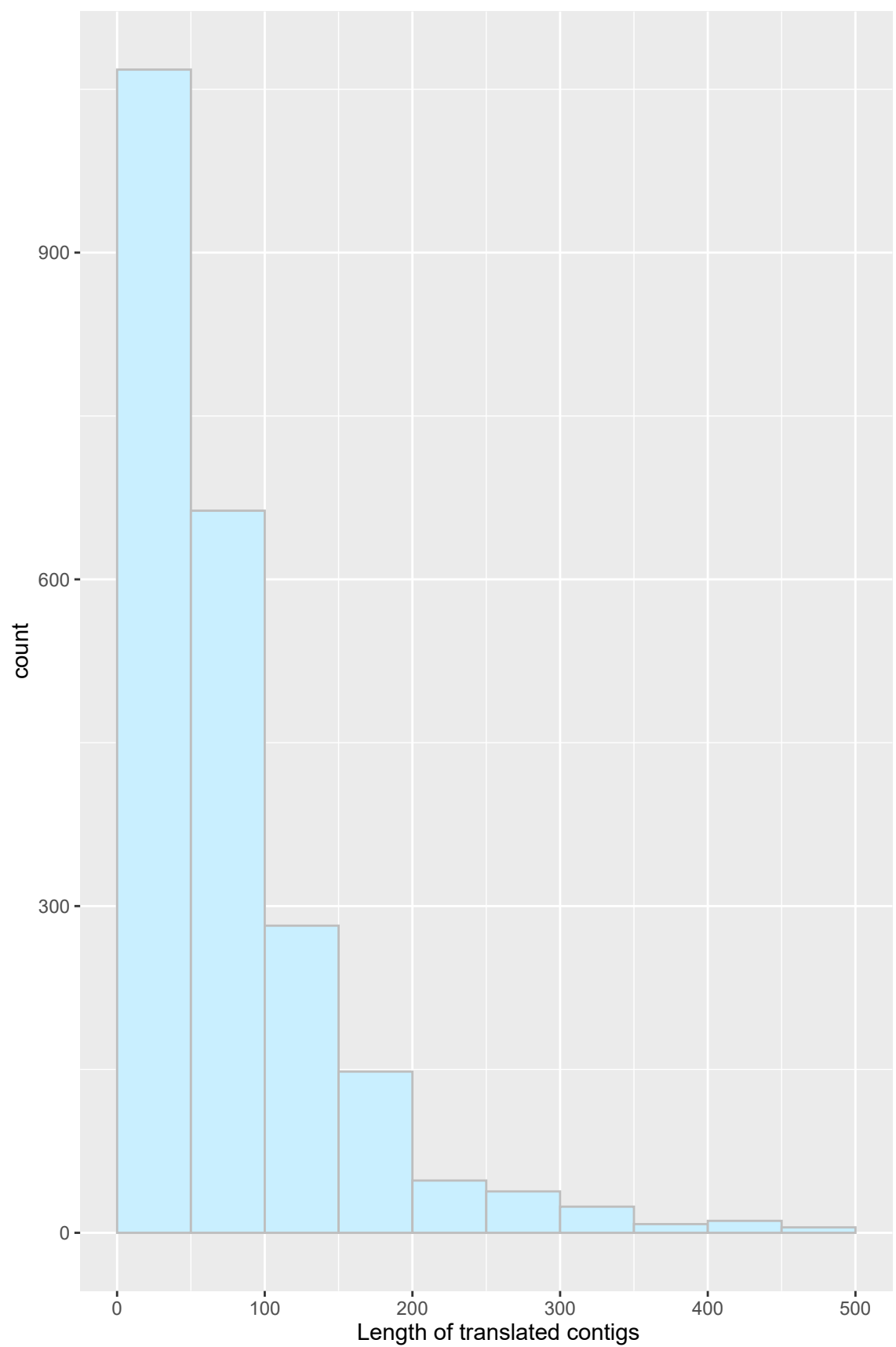

Supplement: Supplementary file 5 [file Image6.pdf]

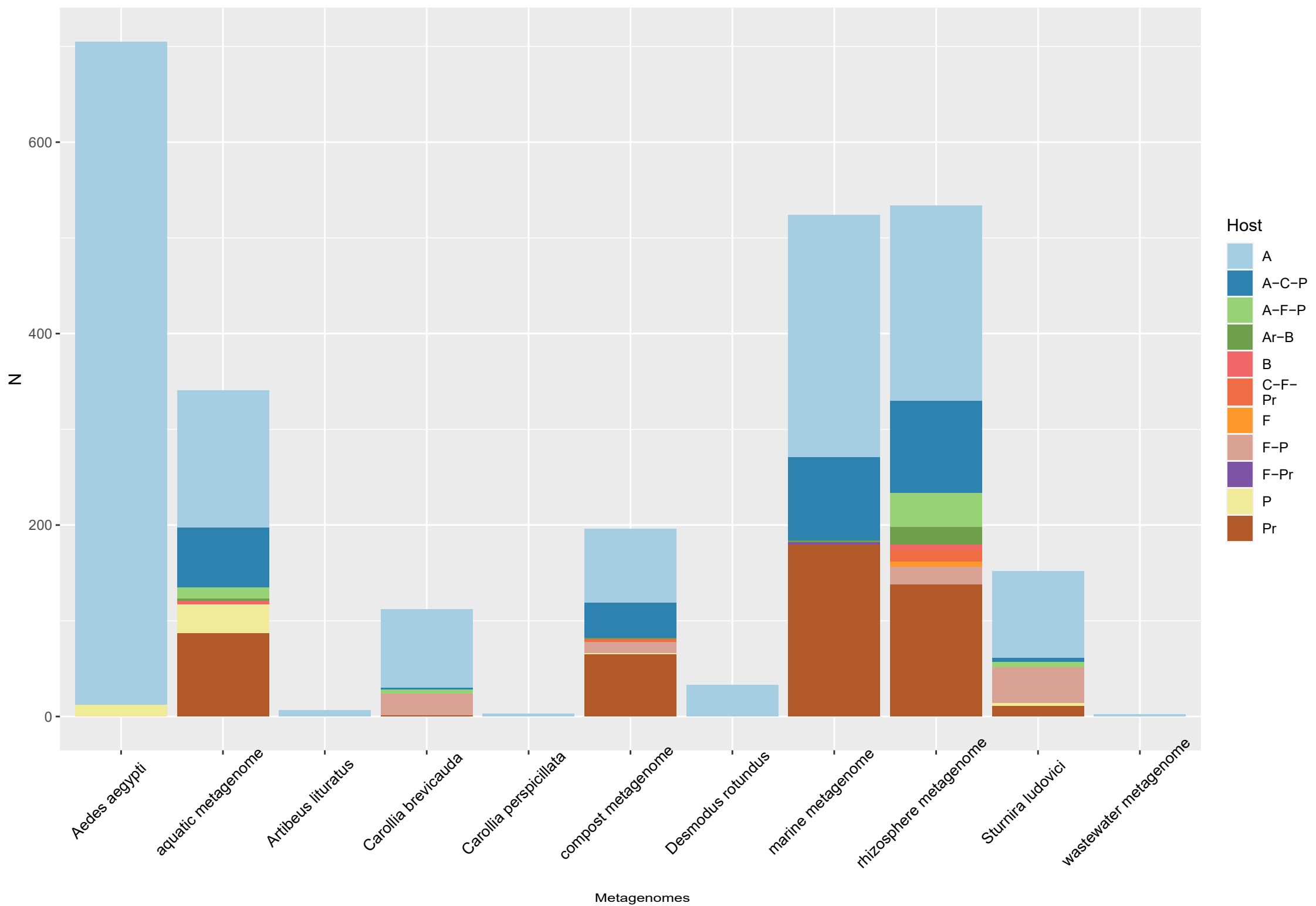

Supplement: Supplementary file 6 [file Image8.pdf]

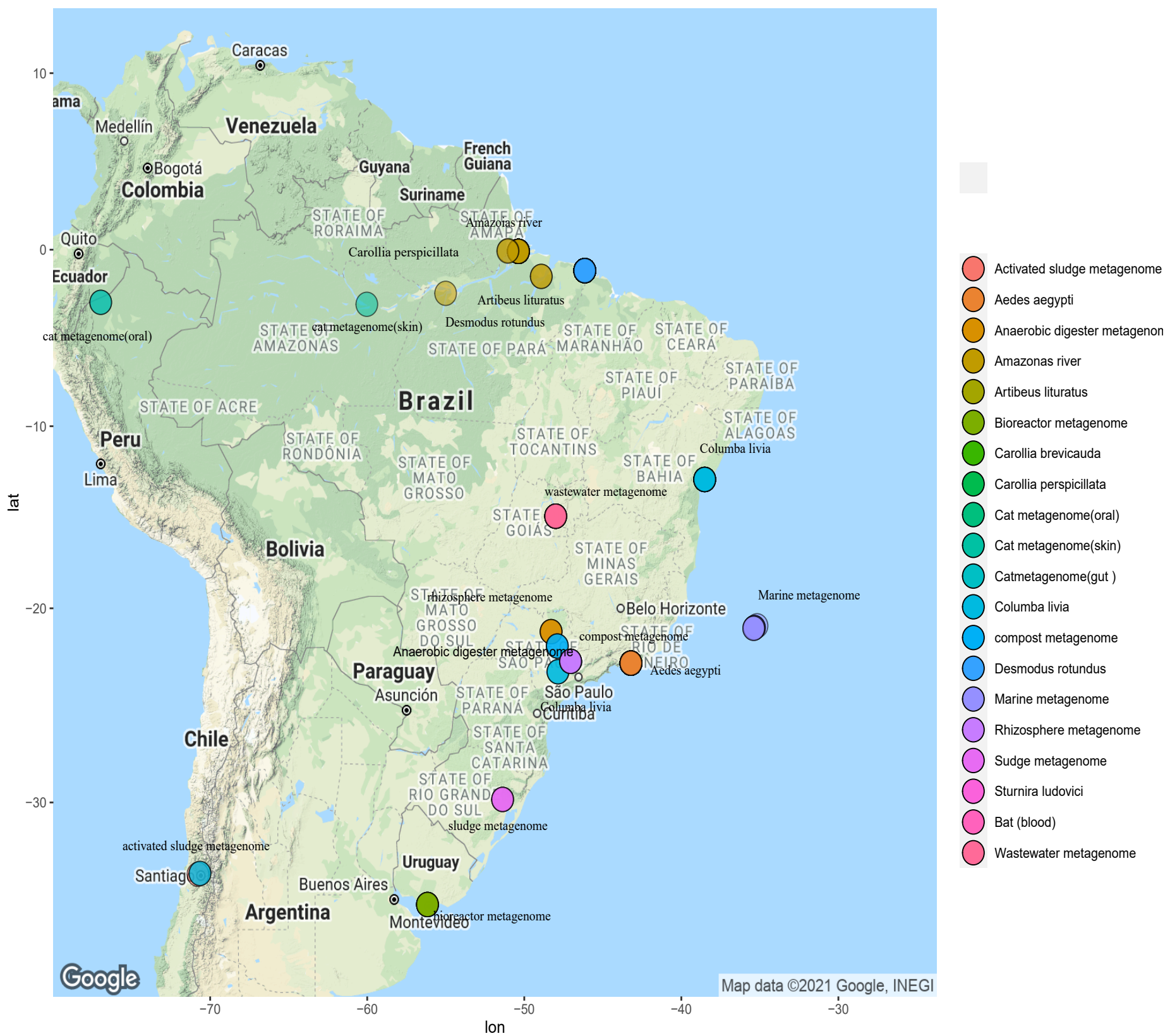

Supplement: Supplementary file 8 [file Image4.pdf]

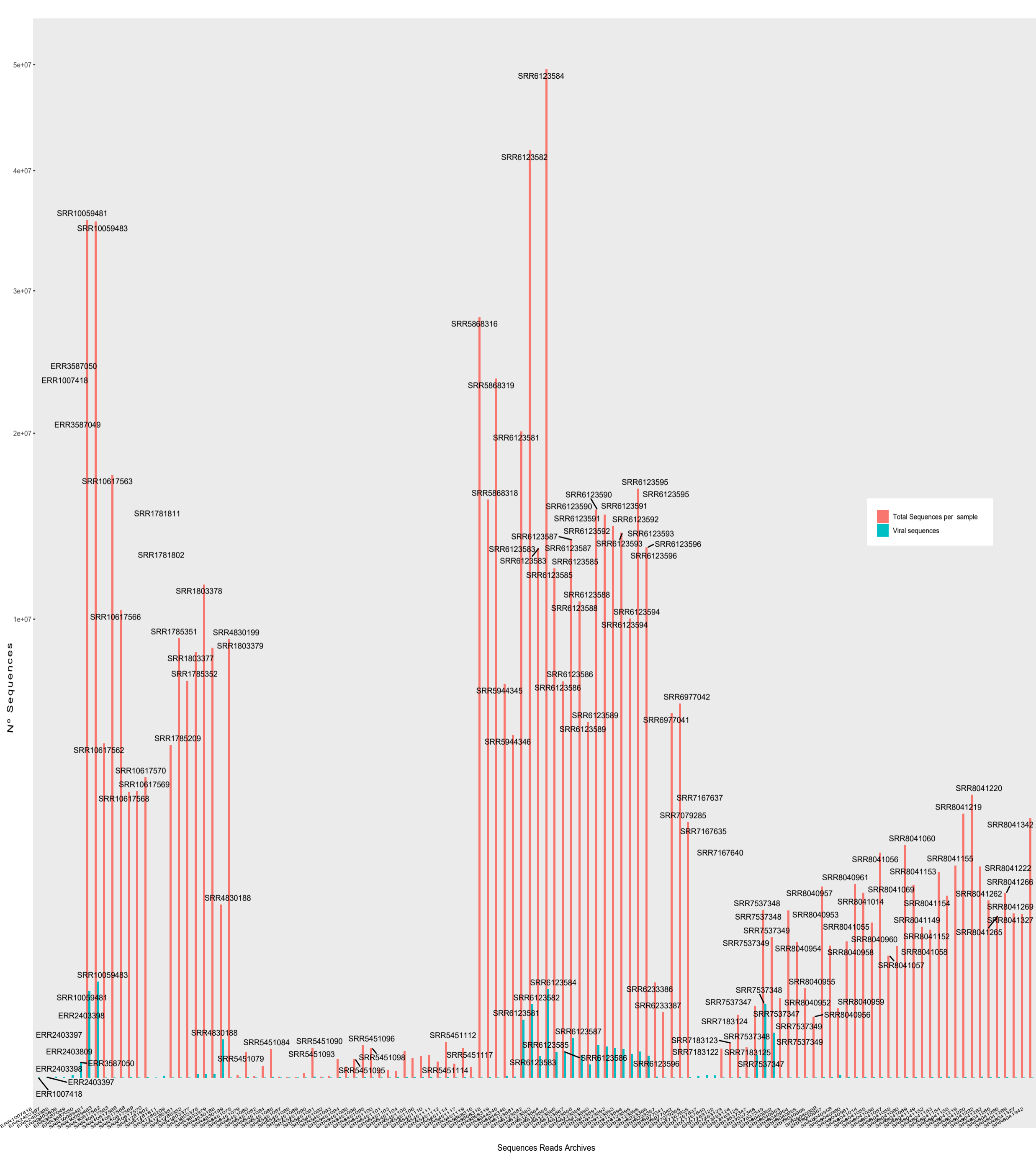

Supplement: Supplementary file 9 [file Image2.pdf]

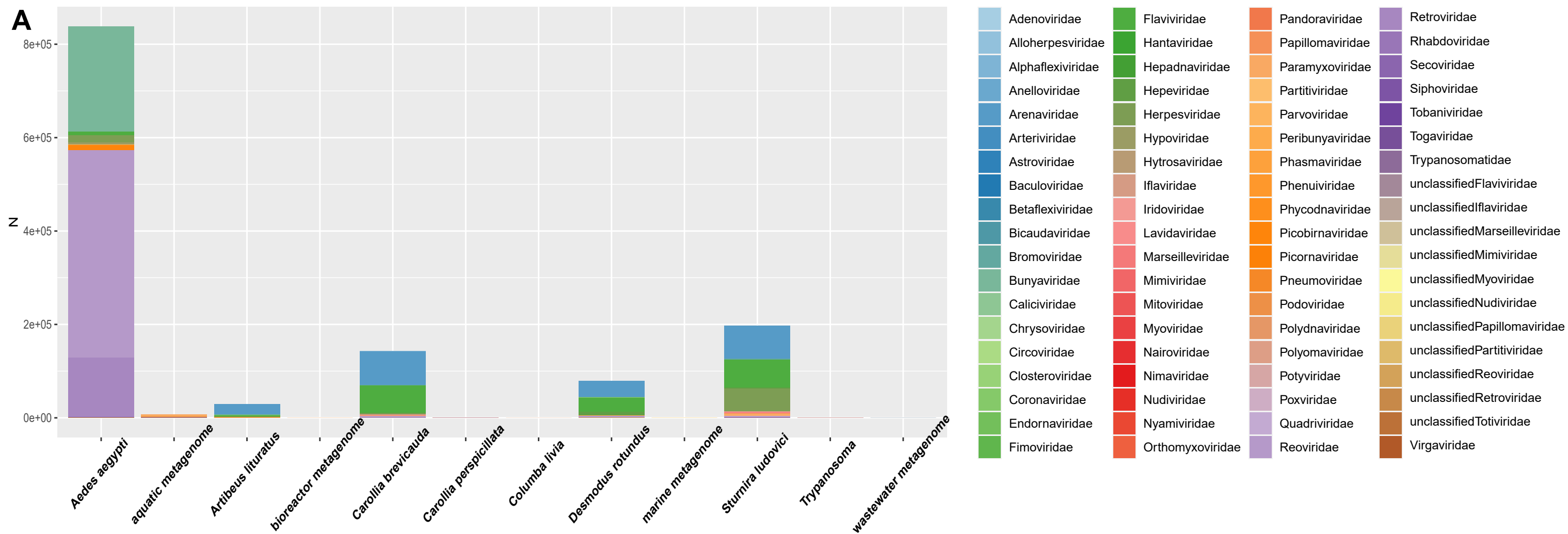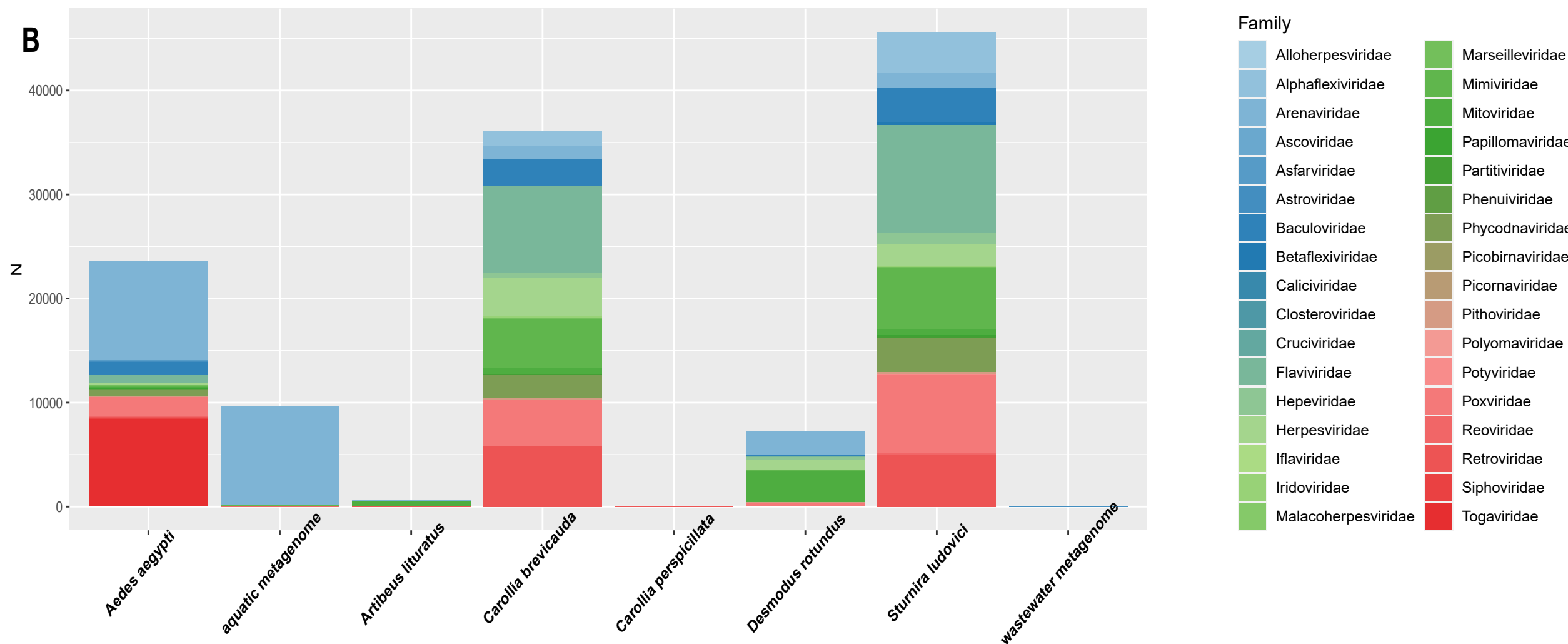

Supplement: Supplementary file 10 [file Image3.pdf]

A)

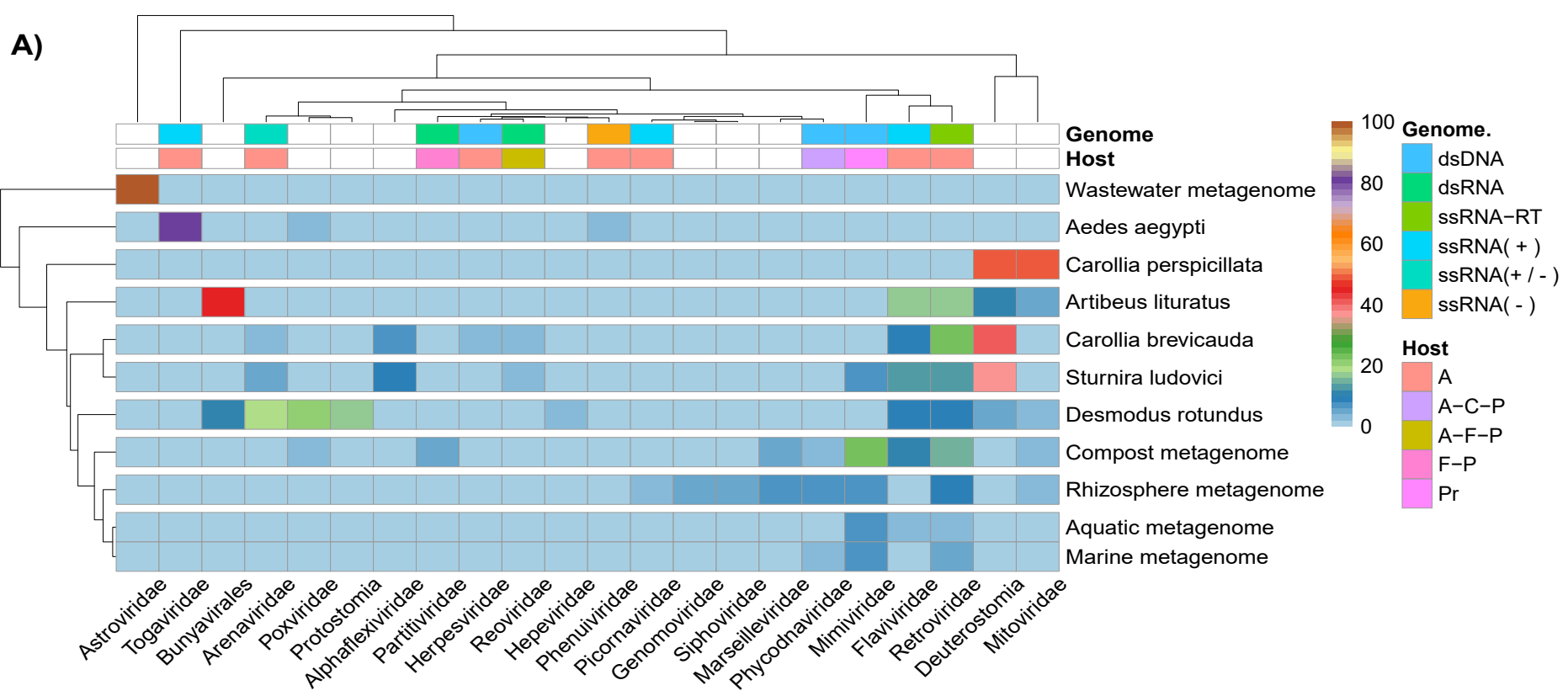

B)

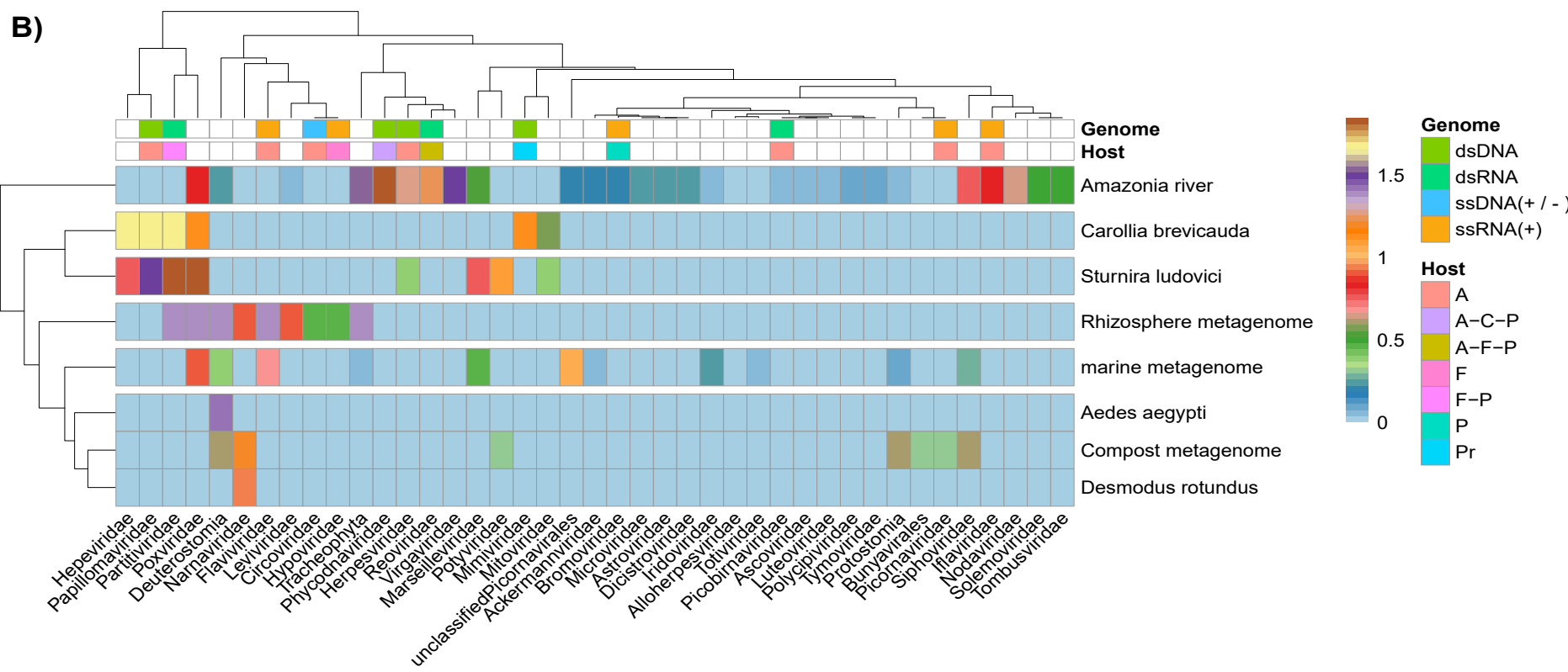

Supplement: Supplementary file 14 [file Image7.pdf]

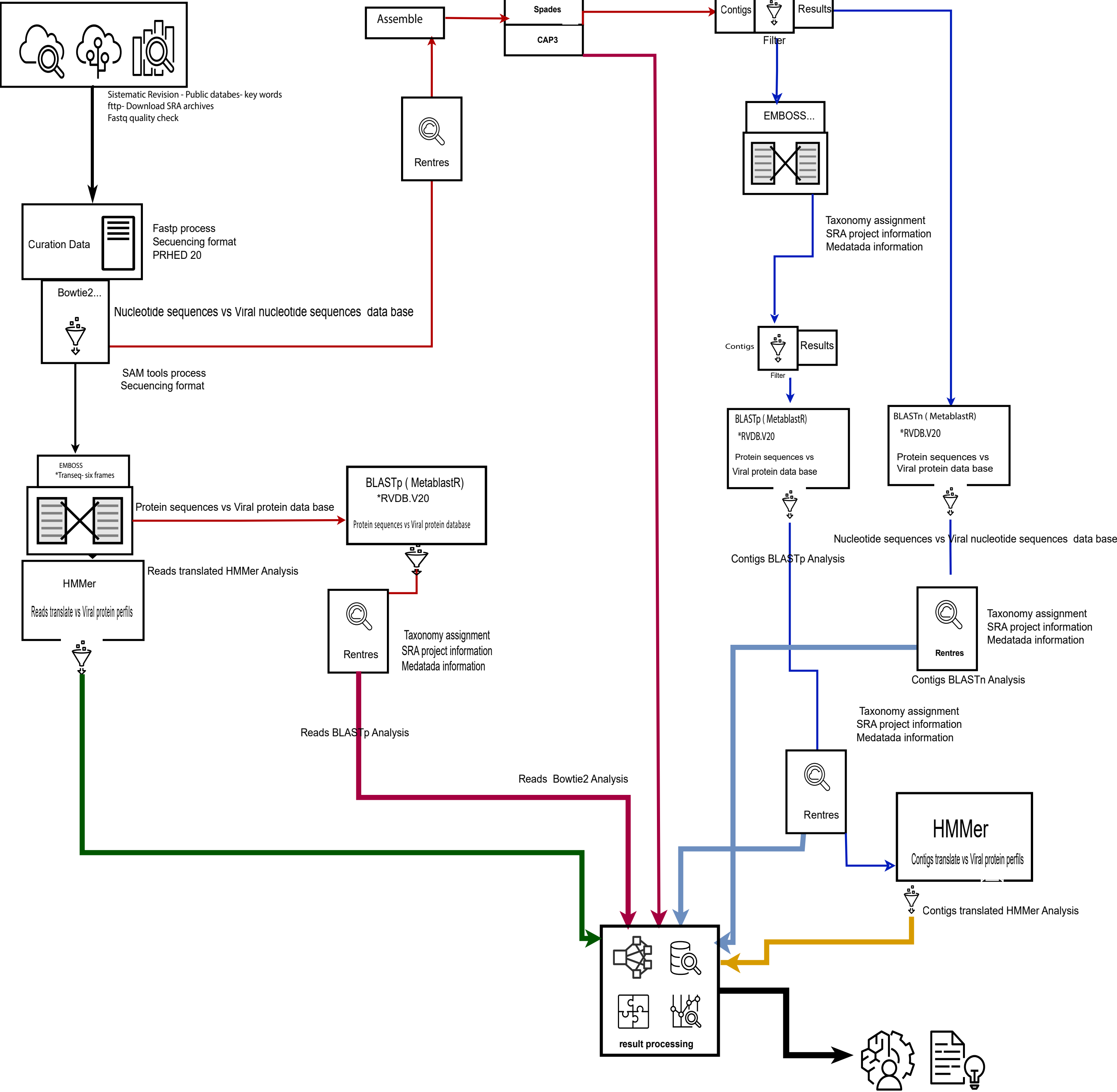

Supplement: Supplementary file 18 [file Image1.pdf]
